# Transcriptomics of single dose and repeated carbon black and ozone inhalation co-exposure highlight progressive pulmonary mitochondrial dysfunction

**DOI:** 10.1186/s12989-021-00437-8

**Published:** 2021-12-15

**Authors:** Quincy A. Hathaway, Nairrita Majumder, William T. Goldsmith, Amina Kunovac, Mark V. Pinti, Jack R. Harkema, Vince Castranova, John M. Hollander, Salik Hussain

**Affiliations:** 1 Division of Exercise Physiology, West Virginia University School of Medicine, Morgantown, WV USA; 2 Mitochondria, Metabolism and Bioenergetics Working Group, West Virginia University School of Medicine, Morgantown, WV USA; 3 Center for Inhalation Toxicology (iTOX), West Virginia University School of Medicine, Morgantown, WV USA; 4 Department of Physiology and Pharmacology, West Virginia University School of Medicine, 64 Medical Center Drive, PO Box 9229, Morgantown, WV 26506-9229 USA; 5 West Virginia University School of Pharmacy, Morgantown, WV USA; 6 Department of Pathobiology and Diagnostic Investigation, College of Veterinary Medicine, Michigan State University, East Lansing, MI USA

**Keywords:** Ozone, Carbon black, Ultrafine/nanoparticle, Inhalation toxicology, Mitochondria, Genomics, Lung, Environmental exposure

## Abstract

**Background:**

Air pollution is a complex mixture of particles and gases, yet current regulations are based on single toxicant levels failing to consider potential interactive outcomes of co-exposures. We examined transcriptomic changes after inhalation co-exposure to a particulate and a gaseous component of air pollution and hypothesized that co-exposure would induce significantly greater impairments to mitochondrial bioenergetics. A whole-body inhalation exposure to ultrafine carbon black (CB), and ozone (O_3_) was performed, and the impact of single and multiple exposures was studied at relevant deposition levels. C57BL/6 mice were exposed to CB (10 mg/m^3^) and/or O_3_ (2 ppm) for 3 h (either a single exposure or four independent exposures). RNA was isolated from lungs and mRNA sequencing performed using the Illumina HiSeq. Lung pathology was evaluated by histology and immunohistochemistry. Electron transport chain (ETC) activities, electron flow, hydrogen peroxide production, and ATP content were assessed.

**Results:**

Compared to individual exposure groups, co-exposure induced significantly greater neutrophils and protein levels in broncho-alveolar lavage fluid as well as a significant increase in mRNA expression of oxidative stress and inflammation related genes. Similarly, a significant increase in hydrogen peroxide production was observed after co-exposure. After single and four exposures, co-exposure revealed a greater number of differentially expressed genes (2251 and 4072, respectively). Of these genes, 1188 (single exposure) and 2061 (four exposures) were uniquely differentially expressed, with 35 mitochondrial ETC mRNA transcripts significantly impacted after four exposures. Both O_3_ and co-exposure treatment significantly reduced ETC maximal activity for complexes I (− 39.3% and − 36.2%, respectively) and IV (− 55.1% and − 57.1%, respectively). Only co-exposure reduced ATP Synthase activity (− 35.7%) and total ATP content (30%). Further, the ability for ATP Synthase to function is limited by reduced electron flow (− 25%) and translation of subunits, such as ATP5F1, following co-exposure.

**Conclusions:**

CB and O_3_ co-exposure cause unique transcriptomic changes in the lungs that are characterized by functional deficits to mitochondrial bioenergetics. Alterations to ATP Synthase function and mitochondrial electron flow underly a pathological adaptation to lung injury induced by co-exposure.

**Supplementary Information:**

The online version contains supplementary material available at 10.1186/s12989-021-00437-8.

## Introduction

According to the World Health Organization, over 4 million deaths annually are attributed to outdoor environmental pollution [1]. Additionally, an increase in air pollution is associated with a tremendous economic burden, a significant increase in disability adjusted life years (DALYs), and an increase in hospitalizations due to exacerbation of pre-existing respiratory and systemic disorders [2–4]. Air pollution is a heterogenous mixture of particulates and gaseous components. Composition of this mixture is highly variable and depends upon a variety of factors including source and atmospheric conditions. These particulate and gaseous air pollution components dynamically shape health outcomes [5]. While air pollution exposures are known to be very heterogenous, most experimental research and current regulations are based on single toxicant exposure. These single toxicant studies fail to account for the potential interactive impacts of the particulate and gaseous components. Recent epidemiological data indicate that gaseous and particulate components interact in shaping the health impacts of air pollution [6]. It is plausible that these interactions are the basis of the observed increases in air pollution associated lung and systemic pathologies despite a significant drop in the mass of air pollution constituents over the past decades [7]. This information is critically needed not only to improve regulatory guidelines but also to enhance the protection of global environmental health.

Ozone (O_3_) is one of the most reactive gaseous components of air pollution and is among six criteria pollutants identified/regulated by the US Environmental Protection Agency (EPA). Ground level O_3_ is generated by photochemical reactions between the ultraviolet component of sunlight and oxides of nitrogen and volatile organic compounds from vehicular emissions. Both epidemiological and experimental evidence confirm the ability of a short term acute O_3_ exposure to aggravate pre-existing disorders [8–10]. Global O_3_ exposure was estimated to be associated with 2,540,000 deaths and a 14% increase in global O_3_ levels is anticipated in the next two decades [2, 11, 12]. Ultrafine/nano carbon black (CB) is widely used as a reinforcing agent in rubber, printing, and leather industries [13]. Global consumption of engineered carbon black is forecasted to reach 19.2 million metric tons, valued at $20.4 billion, by 2022 [14]. CB is classified as a possible human carcinogen (2B) by the International Agency for Research on Cancer (IARC) [15].

CB provides a unique opportunity to study the contribution of the carbon core of particulate matter without interferences from any additives. In addition, there is the possibility of adding additional components as CB particles are excellent carriers of environmental toxicants. A limited number of studies previously addressed particle (PM, diesel exhaust particles, CB) and O_3_ exposure in a sequential manner and identified potentials for deleterious cardiovascular outcomes and increased lung injury and irritation [16–19]. A recent study demonstrated increased lung injury and formation of a fluvic acid-like substance after intratracheal administration of O_3_ reacted CB particles [20]. To date, only a few PM and O_3_ co-exposure studies have been performed, which mainly focused on cardiovascular outcomes and did not address pulmonary inflammatory mechanisms [21–23]. A recent study indicated the potential of ultrafine PM and O_3_ co-exposure to increase lung injury in spontaneously hypertensive rats [24]. These studies clearly suggest a potential for interactive outcomes after particle and gaseous co-exposures.

The application of broad-scale sequencing and “omics” approaches have been used in studies examining the health effects of air toxicants [25–27]. Specifically, transcriptomics provides a useful tool to determine the immediate adaptation, or maladaptation, after a stimulus. In both newborn [28] and adult [29] mice, O_3_ exposure is associated with alterations to the pulmonary transcriptome, which contribute to cellular signaling involving cellular proliferation and differentiation. Maternal exposure to CB has been linked to transcriptomic changes in extrapulmonary organs of progeny, such as the hippocampus [30] and liver [31]. While these studies demonstrate the evident impact of O_3_ and CB on mRNA expression, they are unable to model the interaction of these toxicants in vivo to induce potentially new or more exaggerated cellular responses.

The purpose of this study was to use transcriptomics as a tool to identify unique pathways induced by CB and O_3_ co-exposure (hypothesis generating) and to further assess the validity of these pathways using functional assessments (hypothesis testing). We explored these responses in a single vs multiple exposure scenario and evaluated transcriptomic findings using histological and biochemical methods. We also developed an inhalation co-exposure system with advanced engineering controls and a real-time feedback monitoring system to enable co-exposure inhalation studies that directly model environmental exposure scenarios. A better understanding of the co-exposure-induced unique transcriptional pathways and molecular adaptations in the lungs will aid in development of more realistic and effective public health measures for preventing air pollution-induced pulmonary and systemic pathologies.

## Materials and methods

### Exposure system and aerosol characterization

**Fig. 1:**
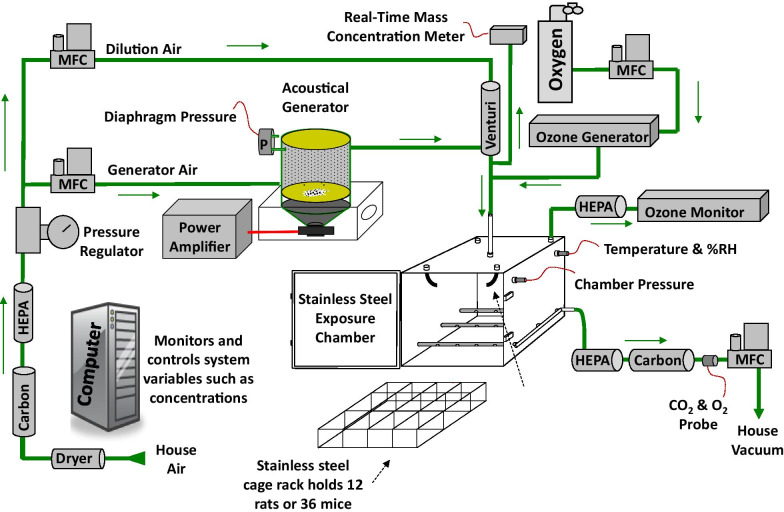
Inhalation exposure system for rodent exposure to air, CB, O_3_, and CB + O_3_ co-exposure. Animals were exposed to air, CB (10 mg/m^3^), O_3_ (2 ppm), or CB + O_3_ for 3 h for either one time or four times (24 h apart) and sacrificed 24 h post last exposure

A whole-body inhalation exposure system was designed to expose up to 36 mice simultaneously to CB aerosols, O_3_, or a mixture of the two toxicants. A diagram of the system is shown in Fig. 1. Ultrafine CB (14 ± 4 nm primary particle diameter) (Printex 90®, a gift from Evonik, Frankfurt, Germany), was placed within a modified high-pressure acoustical generator (HPAG, IEStechno, Morgantown, WV). Acoustical energy deagglomerated and aerosolized the CB material. Compressed air was dried then filtered through HEPA (high efficiency particulate air filter) and charcoal. Clean air was passed through the HPAG and the air-CB aerosol mix was fed into a venturi pump (JS-60 M, Vaccon, Medway MA) that acted as a second stage for particle deagglomeration. A light scattering monitor (DataRAM, pDr-1500, Thermo Environmental Instruments Inc, Franklin, MA) sampled the airstream to estimate the real-time mass concentration of the aerosol. O_3_ was generated by passing clean air or oxygen through a corona discharge O_3_ generator (HTU500AC, Ozone Solutions, Hull, IA).

During co-exposures, the CB aerosol was mixed with the O_3_ before entering the exposure chamber. A 150-L stainless-steel exposure chamber (Cube 150, IEStechno, Morgantown, WV) with mice individually housed in a 36-mouse cage rack was used for all exposures. O_3_ levels were monitored during exposures with a calibrated O_3_ monitor (Model 202, 2B Technologies, Inc., Boulder, CO). Gravimetric mass concentration measurements were conducted over the duration of each exposure. The gravimetric results were used to report results and to continually calibrate the DataRAM. Temperature (20–22 °C), relative humidity (50–70%) (HMT330, Vaisala, Helsinki, Finland), and pressure (Model 264, Setra Systems, Inc., Boxborough, MA) were measured in real-time within the exposure chamber. The exhaust from the exposure chamber was HEPA and charcoal filtered before entering the facility vacuum exhaust. The entire system was contained within a walk-in hood for technician safety. Software was developed (HPAG, IEStechno, Morgantown, WV) to record and control system variables. Analog inputs and outputs were processed with a multifunction I/O device (USB-6343, National Instruments, Austin, TX).

All airflows were regulated with mass flow controllers (MFCs) (Alicat Scientific, Inc., Tucson, AZ). Multiple real-time feedback loops were utilized within the system. The pressure within the HPAG was measured (Model 264, Setra Systems, Inc., Boxborough, MA) and maintained at 0.5″ H_2_O with the generator MFC input airflow to optimize aerosol generation. The pressure within the exposure chamber was held at 0.0″ H_2_O by regulating the exhaust MFC to provide animal comfort and to prevent leaks. The CB aerosol mass concentration was maintained by adjusting the acoustical energy levels based on the DataRAM readings and the O_3_ level was regulated by altering the air/oxygen flow through the ozone generator.

Particle size distributions were measured from the exposure chamber with: 1) an electrical low-pressure impactor (ELPI+, Dakati, Tempera, Finland), 2) an aerosol particle sizer (APS 3321, TSI Inc Shoreview, MN), 3) a scanning mobility particle sizer (SMPS 3938, TSI Inc. Shoreview, MN), and 4) a Nano Micro-orifice Uniform Deposit Impactor (Moudi 115R, MSP Corp, Shoreview, MN). Additionally, aerosols were collected on formvar coated copper grids and the particle surface chemical composition and morphology were inspected with Scanning Electron Microscopy (SEM) (Hitachi S-4700 SEM), Transmission Electron Microscopy (JEOL JEM-2100 TEM) and X-Ray Photoelectron Spectroscopy (XPS) (Physical Electronics PHI 5000 VersaProbe XPS/UPS).

### Murine model

All animal studies, including animal housing, sedation, euthanasia, and experimentation were approved by the West Virginia University Animal Care and Use Committee. These studies conformed to the most current National Institutes of Health (NIH) Guidelines for the Care and Use of Laboratory Animals manual. 8-week-old C57Bl/J6 male mice were purchased from Jackson Laboratory (Bar Harbor, ME) and acclimated at the West Virginia University animal care facility before exposure. All animals were maintained in a room with a 12-h light/dark cycle and provided chow and water ad libitum. Mice were euthanized by intraperitoneal injection of Fatal Plus®. 8-week-old C57Bl/J6 male mice were randomly divided into four groups: 1) sham/air, 2) CB, 3) O_3_, and 4) CB-O_3_ co-exposure. Sham animals were exposed to filtered air. Experimental exposures included 10 mg/m^3^ CB for 3 h, 2 ppm O_3_ for 3 h, or co-exposure to 10 mg/m^3^ CB and 2 ppm O_3_ for 3 h. Exposures were either performed once or daily for up to 4 days. Total animals exposed included: sham/air one exposure (n = 4), CB one exposure (n = 4), O_3_ one exposure (n = 4), CB + O_3_ one exposure (n = 4), sham/air four exposures (n = 8), CB four exposures (n = 8), O_3_ four exposures (n = 8), and CB + O_3_ four exposures (n = 8). The mice were monitored for changes in weight and euthanized 24 h after the final exposure (either one or four) for further studies.

The exposure dose for a single ultrafine CB exposure was chosen to simulate the deposited burden in human lungs after a single 8-h work shift exposure at the current United States Occupational Safety and Health Administration (OSHA) permissible level for CB (3.5 mg/m^3^). In terms of environmental exposure, considering 24 h weighted exposure, this translates to a similar amount of CB deposited in 35 days exposure at the current United States National Ambient Air Quality Standard of 35 µg/m^3^. Environmental sources of CB include fuel combustion leading to release of elemental carbon into the air, release from products such as tires, conveyer belts, rubbers, coatings, and plastics [32–34]. In addition, CB is among the top five highly produced engineered nanomaterials for consumer product applications with production expected to reach approximately 19.2 million metric ton production, worth $13 billion market value by 2022 (carbon black user’s guide) [14]. These applications, which include printer toners, rubber tires, paints etc., also have significant potential of adding inhalable ultrafine carbon into the environment. Previous O_3_ deposition studies demonstrate 4–5 times higher rodent exposure doses translate into similar effective doses in controlled exposures to exercising individuals [35, 36]. Thus, our 2 ppm O_3_ exposure (5 times the 400-ppb dose that induce neutrophilia in humans) is relevant for human exposure extrapolation [36]. Another factor in choosing the O_3_ dose was to be comparable with the current mechanistic understanding that is mainly generated at the similar levels. Interestingly, few recent reports confirm significant lung inflammation at even lower doses and future studies should aim for better dose response comparisons [37–39]. Given that 91% of the world population lives in areas where air quality standards are not met and standards are based on time weighted averages, our exposure conditions are certainly relevant for a human exposure scenario [1].

### CB lung burden quantification

CB lung burden was quantified after a single exposure to estimate deposited lung burden. In brief, air, CB and CB + O_3_ groups were euthanized right after a single exposure and the deposited dose in the lungs was quantified by following a previously validated and published method [40].

### Lung inflammation

Following euthanasia, approximately 1 mL ice cold sterile PBS was instilled through the trachea into the lungs via a syringe three times, obtaining a total broncho-alveolar lavage fluid (BALF) of approximately 3 mL. The cells were counted in the lavage fluid and pelleted by centrifugation at 600 RPM for 5 min at 4 °C and used for Cytospin® 4 (Thermofisher Scientific) preparation for differential counts. Cells were stained in Hema 3 (Fisher Scientific) and differential as well as absolute counts were made. The lavage fluid supernatant was stored at − 80 °C for later investigation.

### Bronchoalveolar lavage analyses

Lavage proteins contents were quantified using a Pierce BCA kit (Thermofisher Scientific) according to manufacturer’s recommendations.

### Lung histopathology and Ki67 immunohistochemistry

Non-lavaged lungs were fixed by intratracheal instillation of 10% neutral buffered formalin for histological analyses and 5-micron-thick paraffin–embedded histological sections were stained with hematoxylin and eosin (H&E) for routine light microscopic examination (VS110, Olympus America). A board-certified veterinary pathologist with experience in respiratory toxicologic pathology of laboratory rodents evaluated the pathological changes without knowledge of individual animal exposure history (blinded manner). Immunohistochemistry was performed on lung tissue sections from formalin-fixed, paraffin embedded, lung lobes to identify nuclear incorporation of Ki67 in bronchiolar epithelial cells undergoing regenerative cell proliferation (1:150, monoclonal rabbit anti Ki67 (SP6), Catalog#275R-16, Millipore Sigma, Burlington, MA). ATP5F1 levels in the tissue sections were also evaluated using IHC (1:100, ATP5F1 polyclonal antibody, Catalog # 15999-1-AP, ProteinTech, Rosemont, IL). Immunohistological slides were counterstained with Gill 2 hematoxylin, scanned and digitized with a slide scanner (VS110, Olympus America) before evaluation.

### RNA Isolation/real-time PCR

RNA was isolated from whole lung homogenate after pulverizing the lung tissues at − 80 °C. Total RNA was isolated using a RNeasy Mini Kit (Qiagen) and reverse transcription was performed using a High-Capacity cDNA Reverse Transcription Kit (Thermofisher Scientific). cDNA was diluted to a working concentration of 10 ng/µl. Real-time PCR reaction was performed in triplicate using the AriaMx Real-time PCR System. Primer sequences are provided in Additional file 1. Each PCR reaction mixture contained Syber Green® 12.5 µl, cDNA 5 µl, Primers 3 µl and Nuclease Free Water 2 µl with a total volume of 22.5 µl. Relative expression levels were measured using a comparative threshold method using Aria Real-Time PCR Software with 18S as internal control. PCR quantification for epithelial alarmins [thymic stromal lymphopoietin (TSLP) and IL-33], inflammatory mediators [interleukin-6 (IL-6), IL-13, tumor necrosis factor-α (TNF-α)] and lung health indicators [club cell protein 10 (CC10) and surfactant protein-c (SFTPC)] was performed. Data were analyzed using the 2^−ΔΔCT^ method [41].

### mRNA sequencing/bioinformatics

RNA was isolated from murine lungs (as described above) and sequenced through the West Virginia University Genomics Core Facility. Libraries were built with the TruSeq® Stranded mRNA Library Prep Kit (Illumina, San Diego, CA) and run on the HiSeq 2500 (Illumina) in 51 bp paired end reads. Fastq files were mapped and quantified using the mapping-based mode of Salmon 1.1.0 [42, 43]. Briefly, a mouse decoy-aware index was created using both the Ensembl primary assembly (release 99) and the transcriptome (release 99). The index was then used to map the paired end reads for quantification, specifying options “--validateMappings” and “--gcBias”. Quantification files were imported into R (v3.6.3) using tximeta [44] and differential gene expression performed through DESeq2 [45]. Visualization packages, such as ggplot2 [46], limma [47], vidger [48], EnhancedVolcano [49], and InteractiVenn [50] were implemented. Kyoto Encyclopedia of Genes and Genomes (KEGG), assessed through pathfinder using 10 iterations [51], was used for ontology [52]. Differential gene expression and gene ontology are provided (Additional file 2).

### Mitochondrial isolations

Mitochondria were isolated from the right lung of C57Bl/J6 male mice using differential centrifugation, as previously detailed [53, 54]. Mitochondria were either immediately used for investigation or stored at − 80 °C in KME buffer (100 mM KCL, 50 mM MOPS, and 500 µM at pH 7.4 in water).

### Electron transport chain (ETC) complex activities

Isolated mitochondria (as described above) were used to assess mitochondrial ETC complex activities. Complex I (NADH dehydrogenase, 340 nm), complex II (succinate dehydrogenase, 600 nm), complex III (cytochrome bc_1_ complex, 550 nm), complex IV (cytochrome c oxidase), and complex V (ATP synthase, 340 nm) were evaluated for substrate metabolism spectrophotometrically. Briefly, this included the substrates NADH (complex I), succinate (complex II), cytochrome C (complex III), reduced cytochrome C (complex IV), and NADH (complex V), coupled with inhibitors of ETC complex activity rotenone (complex I), antimycin a (complex III), and potassium cyanide (complex IV) to determine the activity of each complex. Additional details have been previously described [55]. The Bradford Method [56] was implemented to quantify protein content for normalization of enzymatic activities.

### Western blotting

Immunoblotting was performed using 4–12% gradient gels through MOPS SDS-PAGE, as previously described [54, 55, 57–60]. Normalization of protein content was assessed using the Bradford Method [56]. Primary antibodies utilized in the study included the following: total OXPHOS Blue Native WV Antibody Cocktail (ab110412) (anti-NDUFA9 (complex I, ab14713, Abcam, Cambridge, MA), anti-SDHA (complex II, ab14715, Abcam), anti-UQCRC2 (complex III, ab14745, Abcam), anti-COX IV (complex IV, ab14744, Abcam), and anti-ATP5A (complex V/ATP Synthase, ab14748, Abcam)), anti-ATP5F1 (complex V/ATP Synthase, ab117991, Abcam), and anti-VDAC (#4866, Cell Signaling Technology, Danvers, MA). Goat anti-mouse IgG (H&L) horseradish peroxidase (HRP) conjugate 1:10,000 (Thermofisher Scientific) and goat anti-rabbit IgG HRP conjugate 1:5000 (Abcam) were used as the secondary antibodies. Normalization of protein content was through VDAC expression. Chemiluminescence quantified with Radiance Chemiluminescent Substrate (Azure Biosystems, Dublin, CA), per manufacturer’s instructions and imaged using the G:Box Bioimaging system (Syngene, Frederick, MD). GeneSnap/GeneTools software (Syngene) was used to acquire images. Densitometry was analyzed using Fiji Software (NIH, Bethesda, MD).

### Mitochondrial electron flow

The Seahorse XFe96 Electron Flow Assay was performed as described previously [54]. Briefly, the XFe96 instrument was equilibrated at 37 °C overnight. Isolated lung mitochondria were plated at a density of 2.5 ug per well in the XFe96 culture plate in a volume of 50 uL containing 1X mitochondrial assay solution (MAS) with 10 mM pyruvate, 2 mM malate, and 4 uM FCCP. MAS is composed of: 70 mM sucrose, 220 mM mannitol, 10 mM KH_2_PO_4_, 5 mM MgCl_2_, 2 mM HEPES, 1 mM EGTA, and 0.2% fatty acid free BSA. The XF plate was centrifuged for 20 min at 2000 RSF in a swinging bucket rotor. After centrifugation, 125 uL of MAS was added to each well to bring each well to a final volume of 175 uL. The plate was incubated in a non-CO_2_ incubator for 10 min. The XF cartridge was prepared for injection for ports A (25 uL), B (25 uL), C (25 uL), and D (25 uL). Port A was loaded with rotenone (20 uM), Port B was loaded with succinate (100 mM), Port C was loaded with antimycin-A (40uM) and Port D was loaded with ascorbate (10 mM) and TMPD (1 mM). The cartridge was calibrated by the machine and the assay continued using the protocol developed previously [61]. “Basal” is reported as the mean of the two OCR measurements before rotenone injection for each sample, “Rotenone” is reported as the OCR measurement directly after rotenone injection, “Succinate” is reported as the OCR measurement directly after succinate injection, “Antimycin-A” is reported as the OCR measurement directly after antimycin-A injection, and “Ascorbate/TMPD” is reported as the OCR measurement directly after ascorbate/TMPD measurement. Each group consisted of n = 4, and each sample had 3 technical replicates.

### IPA

The oxidative phosphorylation pathway was generated through the use of IPA (QIAGEN Inc., https://www.qiagenbioinformatics.com/products/ingenuity-pathway-analysis) [62].

### Hydrogen peroxide (H_2_O_2_) quantification

The H_2_O_2_ levels were quantified from whole lung homogenate using Amplex™ Red Hydrogen Peroxide/Peroxidase Assay Kit (Thermo Fisher Scientific, Waltham, MA), following manufacturer’s instructions. Briefly, the lung tissues were pulverized at − 80 °C followed by lysis using RIPA buffer (Thermo Fisher Scientific, Waltham, MA) supplemented with protease inhibitor cocktail (Sigma-Aldrich, St. Louis, MO). The lung proteins were incubated with HRP and Amplex Red dye. The Amplex Red fluorescent dye reacts with H_2_O_2_ to produce the red-fluorescent oxidation product, resorufin. The SpectraMax®iD5 (Molecular Devices, CA) plate reader was used to measure the absorbance at 560 nm after 45 min of incubation time. All final results were normalized to total protein measured using BCA (bicinchoninic acid) assay kit (Thermo Fisher Scientific, Waltham, MA) for quantification [63].

### ATP assay

The ATP content was quantified from frozen whole lung tissue using ATP Assay Kit (Biovision, Inc. Milpitas, CA), following manufacturer’s instructions and previously described protocol [64]. Briefly, frozen lung tissues were pulverized at − 80 °C followed by homogenization using assay buffer provided by manufacturer. Total Protein was quantified using BCA (bicinchoninic acid) assay kit (Thermo Fisher Scientific, Waltham, MA). The standard curve for ATP was generated over a range of 20 nM to 1 nM. The phosphorylated glycerol from lung homogenates was measured at 570 nm using SpectraMax®iD5 (Molecular Devices, CA) plate reader after incubation with the probe for approximately 90 min. All results were normalized to total protein measured using BCA (bicinchoninic acid) assay kit (Thermo Fisher Scientific, Waltham, MA) for quantification.

### Statistics

Statistical differences were inferred using Analysis of Variance (ANOVA) followed by a Tukey’s post-hoc test, a two-sided Student’s t-test, or mixed-effects model, where appropriate using GraphPad Prism® 7.0 software. The mixed-effects model applied Dunnett’s multiple comparison test, with a single pooled variance, for statistical assessments of data containing both duration of exposure and type of exposure as variables. Differences between groups were considered statistically significant if *P* ≤ 0.05, denoted by *. Data are presented as the mean ± standard error of the mean (SEM), when appropriate. For mRNA sequencing, normalized counts > 2 in more than three samples were considered in statistical analyses. Significance for sequencing results were set to a *P* adjusted value (*P*_*adj*_) ≤ 0.05. The false-discovery rate (FDR) was set to 0.05 and statistical testing was performed through the Wald test.

## Results

### Exposure characteristics

**Fig. 2:**
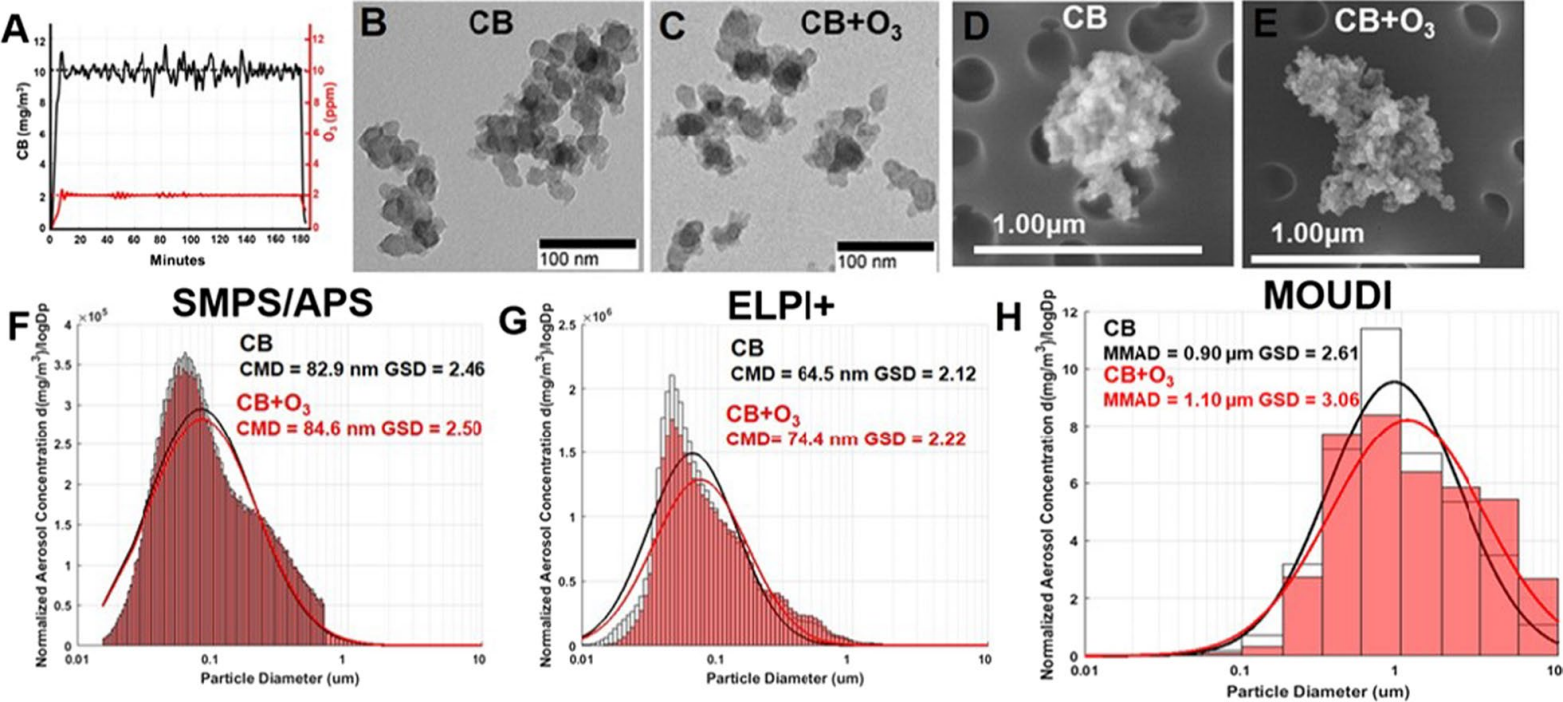
Exposure aerosol monitoring and characterization. **A** Real-time monitoring of CB and O_3_ levels in the exposure chamber during exposure. Representative TEM and SEM images of **B**, **D** CB and **C**, **E** CB + O_3_ aerosol particles collected from inhalation chamber, respectively. Representative figures of aerosol size distribution and log normal distribution fits on data collected using **F** SMPS/APS (Scanning Mobility Particle Sizer/ Aerodynamic Particle Sizer) **G** ELPI + (Electrical Low Pressure Impactor) and **H** MOUDI (Micro-orifice Uniform Deposit Impactor)

Real-time monitoring of CB and O_3_ levels inside the exposure chamber confirmed the generation of stable aerosols (Fig. 2A). The aerosols (CB and CB + O_3_) collected on filters were analyzed for morphology by TEM and SEM (Fig. 2B–E). Concurrent SMPS/APS measurements confirmed that the majority of the particle in the generated aerosols were in the ultrafine/nano range with a count median diameter of 82.9 nm and 84.6 nm with geometric standard deviations of 2.46 and 2.50, respectively for CB and CB + O_3_ aerosols (Fig. 2F). ELPI + measurements, which are based on charge, resulted in count median diameters of 64.5 and 74.4 nm with geometric standard deviation of 2.12 and 2.22, respectively for CB and CB + O_3_ aerosols (Fig. 2G). Aerosol mass based MOUDI measurements indicated mass median diameters of 0.90 and 1.10 µm with a geometric standard deviation of 2.62 and 3.06, respectively for CB and CB + O_3_ aerosols (Fig. 2H). We quantified CB lung burden after a single exposure to be 2.2 ± 0.2 µg/lung at the end of a single exposure both in the case of CB and CB + O_3_ inhalation. This burden translates into one day of human exposure at current OSHA-PEL or 35 days of exposure at the current United States National Ambient Air Quality Standard of 35 µg/m^3^ (considering 24 h weighted exposures).

### Pulmonary inflammation and permeability

**Fig. 3:**
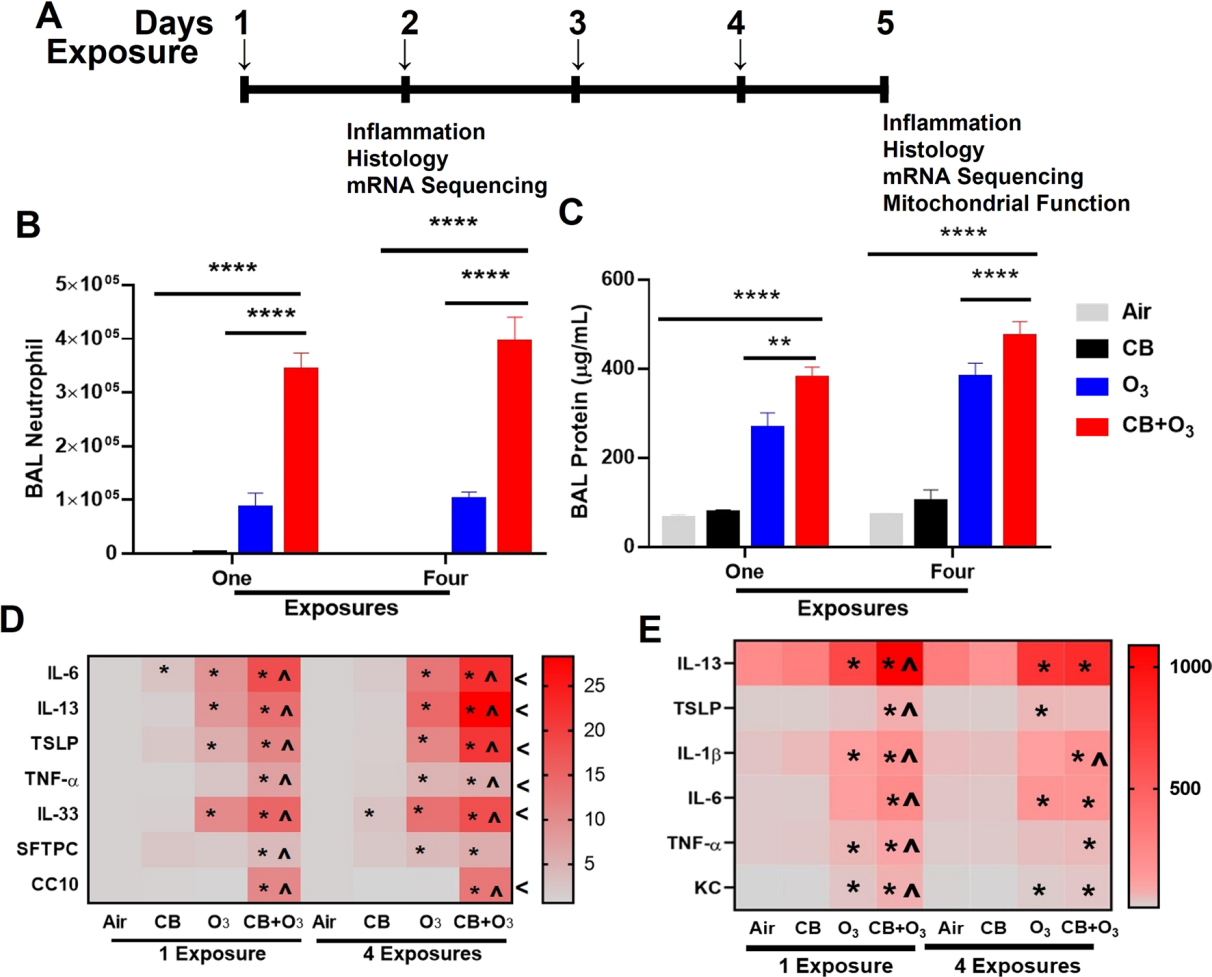
Exposure design and analysis of lung inflammation. **A** Layout of animal exposure experiments. **B** Number of polymorphonuclear leukocytes-neutrophils in bronchoalveolar lavage fluid. **C** Total protein quantification of bronchoalveolar lavage. **D** Real-time PCR analysis of mRNA expression (fold change) in lung homogenates, and **E** Bronchoalveolar lavage cytokine concentrations (pf/mL) by ELISA assay at 24 h post single and four exposures. Animals were exposed to air, CB (10 mg/m^3^), O_3_ (2 ppm), or CB + O_3_ for 3 h for either one time or four times (24 h apart) and euthanized 24 h post last exposure. Data are presented as the mean ± standard error of the mean (SEM) and analyzed by two-way Analysis of Variance (ANOVA) followed by Turkey’s post hoc test. There were n = 4–5 animals per group and per timepoint were used for these analyses. Where *P ≤ 0.05, **P ≤ 0.01, ***P ≤ 0.001, ****P ≤ 0.0001. PCR Analyses (panel D) *denotes significantly different from air/sham, ^ denotes significantly different from O_3_ at same time point, and ^<^ denotes significantly different between day one and four exposures

A layout of the experimental design is presented in Fig. 3A. Both single or multiple exposures to O_3_ and CB + O_3_ induced a significant increase in lung lavage cellularity, as indicated by a significant increase in number of total lavage cells and macrophages (Additional file 3: Fig. S1A–B). A greater and persistent increase in lavage neutrophils was induced by co-exposure compared with O_3_ only exposure (Fig. 3B). Moreover, a significantly greater and persistent increase in lung lavage total protein contents by co-exposure aerosol inhalation and to a lesser extent by O_3_ inhalation further confirmed deleterious impacts in terms of an increase in air-blood barrier permeability (Fig. 3C). Real-time mRNA expression analyses of the lung tissue homogenates further confirmed significant increase in mRNA expression of several markers for injury (SFTPC, CC10), epithelial alarmins (TSLP, IL-33), and inflammatory mediators (IL-33, TSLP, IL-1β, TNF-α, IL-6, IL-13) (Fig. 3D). These mRNA expression studies confirmed that compared to individual exposures, co-exposure aerosols have significantly greater potency to induce transcriptional changes both following single and repeated exposures. Inflammatory cytokine proteins (IL-6, KC, TNF-α, IL-1β) concentration in bronchoalveolar lavage were significantly increased by co-exposure compared with CB and O_3_ single as well as repeated exposures (Fig. 3E). CB inhalation at the tested doses failed to induce an increase in lavage cellularity and total proteins, only inducing mRNA expression of IL-6 after single exposure and IL-33 after repeated exposures.

### Histopathology for CB, O_3_, and co-exposure

**Fig. 4:**
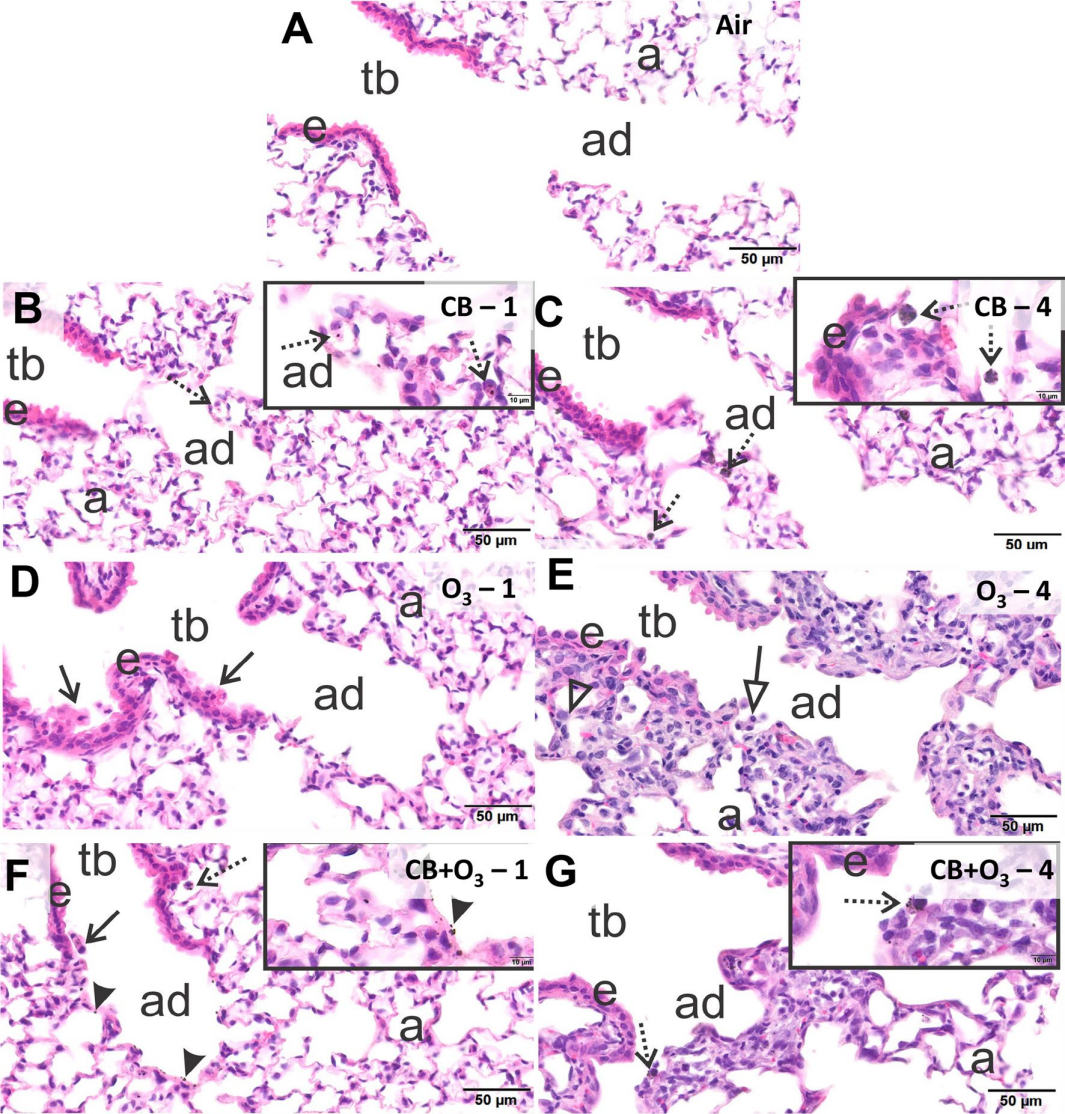
Histopathological analysis of lung tissue injury and inflammation. Light photomicrographs of centriacinar regions in the lungs of mice exposed to **A** filtered air **B** CB, one exposure, **C** CB, four exposures, **D** O_3_, one exposure, **E** O_3_, four exposures, **F** CB + O_3_, one exposure, and **G** CB + O_3_, four exposures. As there was no pathology observed in filtered air single or four exposure groups only single exposure group is presented in the image. Tissue sections were stained with hematoxylin and eosin (H&E) and evaluated by a board-certified veterinary pathologist in a *blinded* manner. N = 3–4 animals per group. tb = terminal bronchiole, ad = alveolar duct, a = alveolar parenchyma, e = bronchiolar airway epithelium, stippled arrows = alveolar macrophages laden with carbon black, solid arrows = exfoliated epithelial cells, open arrow = alveolar macrophages without particles, solid arrowheads = carbon black particles, open arrowhead = type II epithelial cell hyperplasia

Sham mice exposed to filtered air had no pulmonary histopathology after either a single one-day (3 h) (Fig. 4A) or a repeated four-day (3 h/day) inhalation exposure (data not shown). Mice acutely exposed to 10 mg/m^3^ CB had no inflammatory or epithelial lung lesions, but there were conspicuous small black particles in alveolar macrophages most prominent in centriacinar regions, but also widely scattered in the alveolar parenchyma of the lung lobes (Fig. 4B). In addition, a few extracellular CB aggregates were observed in alveolar airspaces. In the lungs of mice exposed for four days to CB, there were slightly more macrophages and more macrophages heavily laden with CB (Fig. 4C). Like in mice acutely exposed to CB, there was no associated inflammatory cell influx, e.g., neutrophils, in the lungs of these animals. In contrast animals exposed for one day to 2 ppm O_3_ had mild multifocal areas of bronchiolar epithelial cell necrosis with exfoliation near some airway branching sites in proximal large-diameter and small-diameter preterminal bronchioles throughout the lung lobes. Similar epithelial necrosis was evident in a few terminal bronchioles (Fig. 4D). After four consecutive days of exposure to O_3_, mice had more pronounced centriacinar lesions consisting of a thinner airway epithelium lining terminal bronchioles that was characterized by low basophilic cuboidal to squamoid, nonciliated, club cells and a noticeable decrease in the number of ciliated cells (Fig. 4E). Proximal alveolar ducts and adjacent alveoli had thickened alveolar septa and hyperplasia of type two alveolar epithelial cells. In conjunction with these later lesions there were also increases in alveolar macrophages/monocytes and a few widely scattered neutrophils (Fig. 4E).

Mice that received a single 3-h co-exposure to O_3_ and CB had multifocal areas of bronchiolar epithelial necrosis in preterminal and terminal bronchioles (lesions similar to those in mice acutely exposed to O_3_) (Fig. 4F). In addition, there were also alveolar macrophages containing CB particles as well as extracellular particles that were predominantly located in centriacinar regions (Fig. 4F). Mice co-exposed to O_3_ and CB for four days had similar centriacinar ozone lesions as described above for mice exposed only to O_3_ for four days (Fig. 4G). In addition, CB-laden macrophages as well as cell free CB were conspicuous in centriacinar regions throughout the lung lobes of these co-exposed animals. Based on the severity of the histological change’s groups were classified from most severe to least severe as follows: CB + O_3_-4 > O_3_-4 > CB + O_3_-1 ≥ O_3_-1 > CB-4 > CB-1. This severity grading was assigned taking into consideration epithelial necrosis, carbon laden macrophage, centriacinar inflammation and epithelial hyperplasia.

### NADPH oxidase pathway changes and hydrogen peroxide levels

**Fig. 5:**
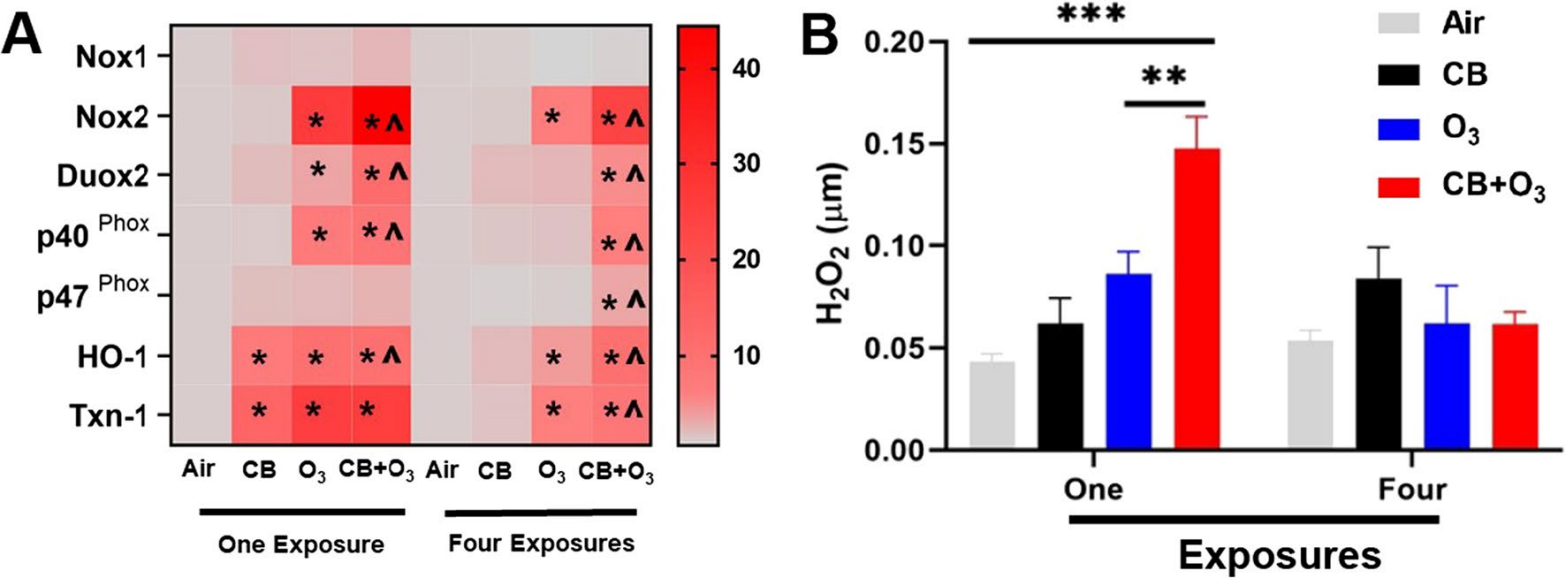
Changes in Nicotinamide Adenine Dinucleotide Phosphate (NADPH) pathway in air, CB, O_3_, and CB + O_3_ co-exposed mice. **A** Real-time PCR analysis of mRNA expression (fold changes) in the lung homogenates **B** hydrogen peroxide (H_2_O_2_) concentration measured by Amplex Red assay in the lung homogenates. For these studies experimental design and exposure concentrations are the same as reported in Fig. 3. Data are presented as the mean ± standard error of the mean (SEM) and analyzed by two-way Analysis of Variance (ANOVA) followed by Turkey’s post hoc test. There were n = 5–7 animals per group and per timepoint were used for these analyses. Where *P ≤ 0.05, **P ≤ 0.01, ***P ≤ 0.001, ****P ≤ 0.0001. PCR Analyses (panel A) * denotes significantly different from air/sham, ^ denotes significantly different from O_3_ at same time point

We evaluated NADPH oxidase pathway related gene expression to evaluate whether this pathway was altered during inhalation exposures. A significant upregulation of mRNA expression for Nox2, Duox2, p40^phox^, and p47^phox^ was observed (Fig. 5A). A trend for increase was observed for Nox1 gene expression. The gene expression of two well-known oxidative stress responsive proteins [heme oxygenase-I (HO-1), and Thioredoxin 1(Txn-1)] further indicated a significant and robust adaptive gene expression response induction after single exposure which was still active after four day exposure. We performed Amplex Red assay to quantify the amount of H_2_O_2_ in the lung tissue and found significantly greater increase by CB + O_3_ exposure after single exposure compared with other individual exposures (Fig. 5B).

### Bioinformatics assessing the duration and exposure of CB, O_3_, and co-exposure

**Fig. 6:**
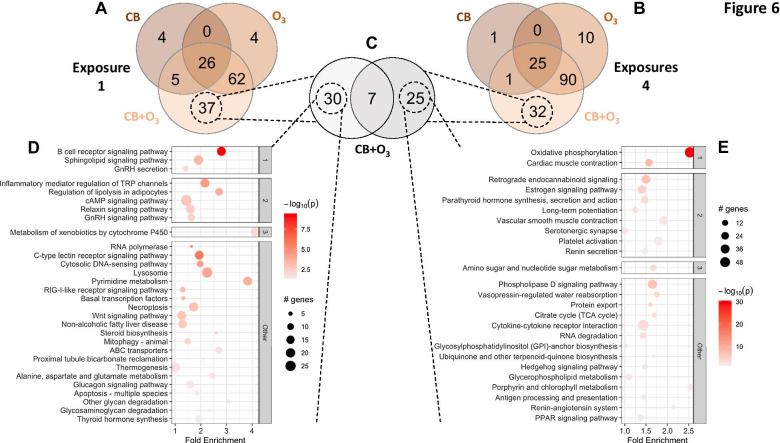
Transcriptomic changes summarized through KEGG gene ontology. Using PathfindR, KEGG gene ontology was applied to the CB, O_3_, and CB + O_3_ groups after **A** single and **B** four exposures. **C** The unique genes identified in the CB + O_3_ group were further stratified to find only unique gene ontologies between the single and four exposures. The unique pathways for **D** single and **E** four exposures are provided. Additional ontology and differential gene expression data is provided in Additional file 1. ID = ID of the enriched term for KEGG ontology, Term Description = description of the enriched term and identified pathway, Fold Enrichment = fold enrichment value for the enriched term (Calculated using ONLY the input genes), Support = the median support (proportion of active subnetworks leading to enrichment within an iteration) over all 10 iterations, Lowest *P*-Value = the lowest adjusted-p value of the given term over all iterations, CB = 10 mg/m^3^ CB exposure, O_3_ = 2 ppm O_3_ exposure, CB + O_3_ = 10 mg/m^3^ CB and 2 ppm O_3_ inhalation co-exposure

To understand the interaction between duration of exposure (i.e., one day versus four consecutive days) and type of exposure (CB, O_3_, or co-exposure) we utilized gene ontology (Fig. 6). The process of integrating gene level changes into pathway analyses first required appreciating how these groups clustered through principal component analysis (PCA) (Additional file 4: Fig. S2A). PCA illustrated that the duration of exposure impacted differential gene expression the most, indicated by the percent variance on the x-axis between groups (Additional file 4: Fig. S2A). Using a differential expression matrix, we further highlighted that four exposures to CB + O_3_ revealed the highest degree of differentially expressed genes when compared to day one exposures of air (5,323), O_3_ (5,532), and CB (6,294) (Additional file 4: Fig. S2B). Additionally, following four exposures, the CB + O_3_ and O_3_ groups showed a significant upregulation of the top 50–100 genes differentially regulated in the analysis (Additional file 4: Fig. S2C, top of heatmap). While we only include a heatmap of the top 500 genes for reference, we have also assessed gene combinations ranging from 50 to 5000 genes (https://github.com/qahathaway/Co-Exposure/tree/main/Heatmaps). These data illustrate that the Day 4 CB-O_3_ and O_3_ groups have very distinct expression profiles that are hierarchically separate from the other groups, regardless of the number of genes assessed.

Next, we needed to understand how the genes were being differentially expressed per group. Volcano plots including all genes for CB, O_3_, and CB + O_3_ at days one and four illustrate the expression profile of all genes analyzed (Additional file 5: Fig. S3). Venn diagrams capturing the number of differentially expressed genes that are shared or unique to the exposures for both single (Additional file 6: Fig. S4A) and four (Additional file 6: Fig. S4B) exposures are provided. The CB + O_3_ co-exposure had the most differentially regulated genes, including the most uniquely altered genes. The uniquely expressed genes in co-exposure for single (Additional file 6: Fig. S4C) and four (Additional file 6: Fig. S4D) exposures are captured in volcano plots. Co-exposure of CB + O_3_ incites a unique transcriptomic response that cannot be captured by an understanding of the toxicology produced from exposure to either of the toxicants alone.

### Unique transcriptomic responses defining co-exposure

The transcriptomic profile of CB + O_3_ is distinctly unique, compared to CB or O_3_ individually, so we wanted to investigate if these regulatory changes were indicative of changes in cellular pathways. Gene ontology through KEGG summarized the differential gene expression data into a more comprehensible assessment of what alterations occur at single (Fig. 6A) and four (Fig. 6B) exposures, represented as Venn diagrams. These Venn diagrams illustrate how 37 (CB + O_3_, one exposure) and 32 (CB + O_3_, four exposures) cellular pathways are unique in co-exposure. We further examined the similarities/dissimilarities of the unique co-exposure pathways (Fig. 6C) and show each of the non-overlapping pathways for single (Fig. 6D) and four (Fig. 6E) exposures. We also provide the overlapping ontologies of all groups in a 6-way Venn diagram (Additional file 7: Fig. S5A) to allow for further appreciation of the stratification of the groups, as well as the 3 pathways that overlap between all groups, and a few of the representative genes (Additional file 7: Fig. S5B). Additionally, all differential expression and gene ontology data are provided (Additional file 2).The pathways represented in Fig. 6D, E reveal a variety of different functions in the cell, so we focused more specifically on the top (lowest *P*-value) pathways. Following single exposure, B cell signaling pathways were altered, highlighting the importance of immune processes engaged during pulmonary insult from xenobiotic particles (Fig. 6D). Interestingly, after four days of exposure, mitochondrial oxidative phosphorylation was the top pathway (Fig. 6E). While activation of immune function is commonly associated with inhaled toxicants, changes in pulmonary metabolism suggest a unique mechanism for adaptation/maladaptation to sustained particle inhalation.

### Lung proliferation after CB-O_3_ co-exposure

**Fig. 7:**
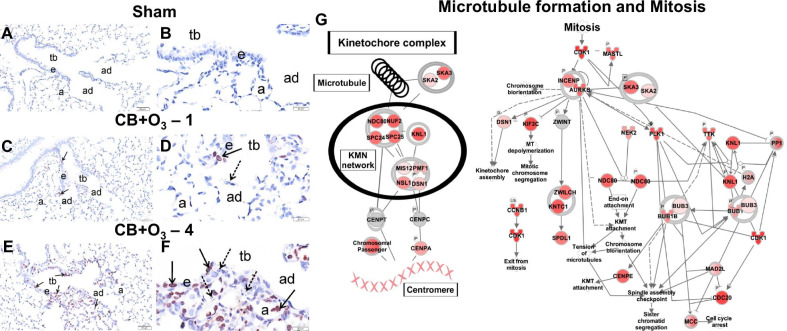
Ki67 proliferation index and genes corresponding to proliferation. Light photomicrographs, at low and high magnifications, of centriacinar regions of the lung from mice exposed by inhalation to **A**, **B** filtered air, **C**, **D** CB-O_3_ for one day, or **E**, **F** CB-O_3_ for four days. Lung tissue sections were immunohistochemically stained for Ki67 (brown chromagen in nuclei of epithelial cells undergoing DNA synthesis, solid arrows) and counterstained with hematoxylin. Stippled arrows, aggregates of carbon black particles; *tb* terminal bronchioles, *ad* alveolar ducts, *e* bronchiolar epithelium, *a* alveoli. (n = 3). **G** The top canonical pathway in Ingenuity Pathway Analysis (IPA) modified in co-exposure at day four (n = 3). Red indicates an increase in expression of the gene in the transcriptomic analysis. The darker the shade of red, the greater increase in expression. Day 1 (n = 4, each group) and Day 4 (n = 3, each group). Number following the exposure condition denotes number of times (either one or four) animals were exposed. Animals were euthanized and analyzed 24 h post single or four exposures. *tb* terminal bronchiole, *ad* alveolar duct, *a* alveolar parenchyma, *e* bronchiolar airway epithelium, *DE* differentially expressed genes

With transcription of mitochondrial proteins altered, we wanted to know if this was part of a general response initiated by increases in cellular replication. Proliferating cells in the lung sections were immunostained with Ki-67. We observed minimal proliferative response in air exposed mice lungs (Fig. 7A, B), mild Ki67 staining in mice co-exposed to CB and O_3_ for one day (Fig. 7C, D), and marked Ki67-stained nuclei in mice similarly co-exposed for four days (Fig. 7E, F). Interestingly, when looking at all pathways, unique or common, Ingenuity Pathway Analysis (IPA) revealed that the most significantly expressed (P ≤ 0.001) canonical pathway after four exposures to CB + O_3_ was related to microtubule formation and mitosis (Fig. 7G). Agreeing with the histological presentation of the group, the IPA schematic reveals likely molecular players involved in modulating the proliferation response following co-exposure.

### Mitochondrial electron transport chain (ETC) and electron flow

**Fig. 8:**
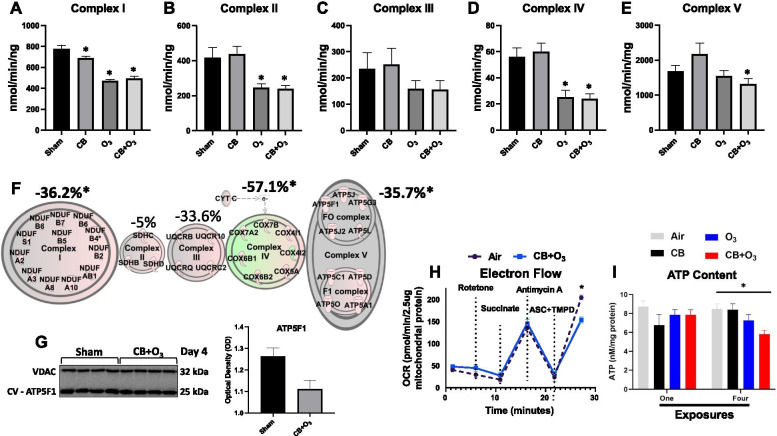
Mitochondrial gene expression and function after four exposures. Mitochondrial electron transport chain maximal activities defined as the amount of substrate consumed per minute per ng or mg of protein for **A** complex I, **B** complex II, **C** complex III, **D** complex IV, and **E** complex V at day four exposure (n = 4, each group). **F** Using Ingenuity Pathway Analysis (IPA), mitochondrial genes that were differentially expressed in the oxidative phosphorylation pathway are displayed for co-exposure. Genes colored red have statistically increased transcription, genes colored green have statistically decreased transcription, and genes colored grey have no change in transcription, compared to the air control group. The shape of the genes is not indicative of function or pathology. Coloring of the entire complex, i.e. complex I, II, III, IV, and V, indicates global increase/decrease of expression of the entire complex. **F** The maximal activities of ETC complexes I–V are displayed above each of the corresponding complex units. **G** Western blot of ATP5F1 and quantification. **H** Electron flow was measured in co-exposure compared to the sham group for complexes I–IV. **I** Total ATP content was quantified by day and group for CB, O_3_, and CB + O_3_ groups. Day 1 (n = 4, each group) and Day 4 (n = 4, each group). Differences between groups were considered statistically significant if *P* ≤ 0.05, denoted by *. Data are presented as the mean ± standard error of the mean (SEM), when appropriate. Sham—4 = filtered air exposed for 4 days, CB—4 = 10 mg/m^3^ CB exposure for 3 h repeated four times (24 h apart), O_3_—4 = 2 ppm O_3_ exposure for 3 h repeated four times (24 h apart), CB + O_3_—4 = 10 mg/m^3^ CB and 2 ppm O_3_ inhalation co-exposure for 3 h repeated four times (24 h apart), *OCR* oxygen consumption rate

CB + O_3_ co-exposure over four days results in an increased transcription of mitochondrial genes (Additional file 2), so we wanted to understand how this correlates with mitochondrial function. ETC maximal activities were measured for each complex (Fig. 8A–E). After four exposures, alterations were seen in complex I with CB, O_3_, and CB + O_3_ (Fig. 8A), complex II with O_3_ and CB + O_3_ (Fig. 8B), none in complex III (Fig. 8C), complexes IV with O_3_ and CB + O_3_ (Fig. 8D), and ATP Synthase with CB + O_3_ (Fig. 8E). Using IPA, we illustrated genes involved in the mitochondrial ETC/oxidative phosphorylation pathway for CB + O_3_ and overlayed this information with ETC activity represented as percent change from the control group (Fig. 8F). After four days of exposure, CB + O_3_ co-exposure resulted in differential expression of 35 genes in the mitochondrial ETC pathway (Fig. 8F).

Observing changes in both the RNA expression and mitochondrial function of the mitochondrial ETC, we next broadly evaluated protein content of the complexes (Fig. S6A). Examining NDUF9A (complex I), SDHA (complex II), UQCRC2 (complex III), COXIV (complex IV), and ATP5A (complex V) protein content, obeserving no changes between the CB + O_3_ and air groups (Additional file 8: Fig. S6B–F). Additionally, we examined if mRNA expression was representative of protein expression for transcripts known to be altered. Immunoblotting of an ATP Synthase subunit, ATP5F1, in isolated mitochondria revealed decreased content in the CB + O_3_ group (Fig. 8G), in contrast to an increased mRNA expression (Fig. 8F). We further performed IHC on the lung tissue for ATP5F1 which indicated an increased expression in the lung areas/cells showing significant alterations such as bronchiolar epithelium and weaker expression was noted in alveolar macrophages (Additional file 9: Fig. S7). This discrepancy pointed towards a potential import issue for this protein into the mitochondria. We further evaluated ATP Synthesis in the CB + O_3_ co-exposure group, assessing electron flow through complexes I–IV to identify if mitochondrial coupling was also dysfunctional (Fig. 8H). The flow of electrons was significantly diminished through complex IV in the co-exposure, as illustrated by a decreased oxygen consumption rate (OCR) when ascorbate and TMPD were added. Additionally, the total ATP content of the lung was significantly decreased after four exposures in the CB + O_3_ group (Fig. 8I). While co-exposure increases expression of mitochondrial transcripts, an imbalance in protein production and a decrease in electron flow efficiency of mitochondria are present.

## Discussion

This study validated interactive transcriptomic alterations induced by inhalation co-exposure to CB and O_3_. Our approach involved first identifying which cellular pathways were significantly impacted following co-exposure, *i.e.,* transcriptomics, followed by testing the validity of these initial findings, *i.e.,* through biochemical, histological, and molecular assessments. In examining the uniquely expressed transcripts after inhalation co-exposure, we identified and validated two critical pathways: mitochondrial oxidative phosphorylation and cellular proliferation. A decrease in mitochondrial function is likely a crucial factor in driving the maladaptive response following co-exposure. This study has significant merit compared with the published literature as this is the first detailed evaluations of unique transcriptomic signature of a particulate and gaseous inhalation co-exposures.

We intentionally decided to use CB and not black carbon (BC) or the particulate matter (PM) collected from environmental sampling. This choice was made since, 1) composition of PM varies significantly over a given period depending upon factors, such as, season, temperature, humidity, source of combustion, and other environmental contaminants *e.g.,* endotoxin, pollen etc., and 2) BC is an already modified carbon material after interacting with O_3_, NOx, and hydrocarbons from combustion [65]. Both materials, while environmentally relevant, cannot simulate individual interactions between different constituents. Indeed, a recent study confirmed that interaction with O_3_ modifies the composition of PM [6]. Our model simplistically confirms the potential of interactions between the ultrafine particles of CB (a low toxicity low solubility material) and O_3_, and thus provides basis for further research by adding additional constituents of air pollution.

Histology and inflammation data clearly indicate the increased toxic potential of co-exposure. While these data failed to provide unique alterations induced by co-exposure, they confirmed significantly greater potential for inhalation co-exposure to induce lung injury and inflammation. In addition, these results are in line and confirm previous rodent studies and human controlled exposure studies for O_3_, indicating lung inflammation and injury [36, 66, 67]. A significant increase in lung PMN/neutrophil levels after a single co-exposure was further aggravated after multiple exposures. Interestingly, in the case of O_3_, multiple exposures did not further enhance this response. It is not surprising, as previous studies show that respiratory alterations, such as neutrophil accumulation, to acute ozone exposure paradoxically resolve during multiple exposure scenarios due to a failure in the mechanisms leading to accumulation of neutrophils at the site of inflammation [68, 69]. Conversely, an obvious synergistic accumulation of neutrophils at both times is evident for CB + O_3_ co-exposure. This indicates that the mechanisms that led to an impaired increase in neutrophils in the case of O_3_ exposure are no longer effective [70].

Increased expression of epithelial alarmins (IL-33 and TSLP), inflammatory mediators (IL-6, TNF-α, IL-13, IL-1β) and lung injury/health markers (CC10, SFTPC) clearly indicate the potential of co-exposure to induce these signaling pathways. CC10 and SFTPC are not only markers of unique cell families in terminal bronchioles and alveoli but are also associated with a variety of pulmonary disorders ranging from interstitial lung diseases to cancer and environmental exposures [66, 71–74]. A previous study demonstrated serum CC10 levels as a sensitive biomarker of O_3_-induced changes in the airway epithelium [71]. Interestingly, we only observed altered gene expression in the CB + O_3_ co-exposure group at 24 h post exposure. Since the current study focused on mRNA expression and employed a different time point and ozone exposure concentration, it is plausible that the observed response is a result of co-exposure-induced altered kinetics for CC10 gene expression. Inflammatory cytokine proteins (IL-6, KC, TNF-α, IL-1β) concentration measurement in bronchoalveolar lavage by ELISA confirmed significantly greater potentials of co-exposure to induce cytokine production.

Mitochondrial functional impairments, such as decreases in bioenergetics, can be attributed to changes in coupling efficiency, ETC integrity, substrate availability, and other similar mechanisms. Mitochondrial function is known to be altered following CB and O_3_ exposure. CB exposure is linked to mitochondrial dysfunction through the inhibition of mitochondrial fission/fusion [75] and increased mitochondrial damage (observed as cytochrome c release) [76]. O_3_ exposure is also known to cause mitochondrial damage, through damaging mtDNA in vascular mitochondria [77], but has also been identified in regulating bioenergetics [78]. Valdez et al. indicated that mitochondrial ETC complex activities in the brain are decreased following pulmonary O_3_ exposure, with diminished activity of complex IV as one of the primary findings. Our results further support the concept that O_3_, as well as CB + O_3_, inhalation exposure results in decreased complex IV activity.

Contrary to the individual CB or O_3_ exposures, co-exposure induces a greater effect with a unique transcriptomic profiles of genes involved in the ETC; this is highlighted by the number of ETC genes differentially expressed in CB (0), O_3_ (2), and co-exposure (35). Although both the O_3_ and CB + O_3_ groups show a reduction in Complex I and IV activity, the co-exposure group uniquely modifies ATP Synthase function. Further, a disconnect exists between mRNA expression and mitochondrial protein levels for ATP Synthase following co-exposure, represented by increased mRNA expression of ATP5F1, with reductions in actual protein content. Contrary to this, we found significant increases in the levels of ATP5F1 in IHC. This suggests that the reduced activity of ATP Synthase is likely due to both the decreased efficiency of electrons travelling through the ETC, identified through electron flow, as well as a disruption in import/integration of subunits for ATP Synthase, such as ATP5F1 into the mitochondria. Observation of such defect in mitochondrial import of proteins is not surprising as we previously demonstrated such defects in mitochondrial protein import in diabetic heart [54, 79]. We only evaluated ATP5F1 levels and did not analyze the entire ATP synthase complex, it is possible that other protein constituents of the ATP synthase complex were also affected by the co-exposure. Using broad scale proteomic methodologies, our collaborators have observed changes in multiple protein constituents in electron transport chain complexes following pathological insults, which likely, in aggregate precipitate functional compromise in the complex [53, 54, 80]. It should be noted that in our study, ATP levels and ATP synthase activity were also decreased in case of the co-exposure, suggesting that the loss of ATP5F1, may be partly responsible for the functional changes observed.

In order to clarify a potential link between the inflammatory response and mitochondrial function, we studied NADPH oxidase system alterations. NADPH oxidase system is among the best described pathways connecting inflammatory response and mitochondrial dysfunction through reactive oxygen species (ROS) production [81–85]. Real time PCR has recently been recommended as the method of choice for initial screening of NADPH pathway [86]. Our results clearly indicated increased mRNA expression of Nox2 as well as Duox2 subunits as well as adaptor proteins P40 ^phox^, and P47 ^phox^. While Nox2 is a well-known for its expression in function in phagocytes, Duox2 is highly expressed by the epithelial cell in the lungs [81, 87, 88]. This increase further confirm exposure induced ROS production pathway activation goes beyond the infiltrating leukocytes and point towards a tissue level biochemical/pathologic alteration. Moreover, our histology data indicate an aberrant repair process with hyperplastic and regenerating epithelia which have known/well established mitochondria activity change implications [89]. These clearly point towards a tissue level change rather than just infiltration of leukocytes. These findings of increased ROS production are in line with our recently published immuno spin-trapping data which also confirmed presence of oxidants in macrophages as well as epithelial cells in the lungs after similar exposure ([90]. NOX derived ROS play an important role in regulating NLRP3 inflammasome (reviewed in [81]). We demonstrate an increase in IL-1β levels (Fig. 3E) which is a typical NLRP3 downstream cytokine. Increase H_2_O_2_ levels after single exposure point towards oxidant generation being an early event followed by mitochondrial damage. Taken together with our recently published results demonstrating a key role of oxidants in lung inflammation and lung function decline through production of thymic stromal lymphopoietin further consolidates the key role of enhanced ROS production in inflammatory responses after CB + O_3_ co-exposure ([90]. It is plausible to hypothesize that this ROS production led to/amplify the mitochondrial damage response that was significantly greater after multiple exposures. It is important to note that mitochondrial damage is known to play a critical role in the pathogenesis of multiple pulmonary disorders which also are exacerbated by the air pollution exposures [91–96].

## Conclusions

In the current study we evaluated the impact of CB and O_3_ co-exposure on the transcriptomic profile of the lungs. By implementing an acute (one day) and sustained (four consecutive days) exposure model, we effectively captured the progressive adaptions that CB, O_3_, and co-exposure produce in the lungs. Co-exposure provokes an exaggerated response that cannot be accounted for based on the individual profiles of the toxicants present. This unique response is best characterized by decreased mitochondrial ETC function, specifically at ATP Synthase. Future assessments will be critical in accurately characterizing the longitudinal effects of particulate matter and ozone on mitochondrial function in the lungs.

## Supplementary Information

**Additional file 1. Raw data values used for generation of heat maps for real time PCR,  ELISA, and primer sequences.**

**Additional file 2. Differential expression analyses.**

**Additional file 3. Fig. S1**: Bronchoalveolar lavage total cells and macrophages depicting increased potency of CB + O_3_ co-exposure to induce lung inflammation after (**A**) 1 Day exposure (n = 5–7) and (**B**) 4 Days exposure (n = 5–7) compared to filtered air, CB (10 mg/m^3^) and O3 (2 ppm). Data are presented as mean ± SEM of n = 5–7 mice per group and analyzed by two-way analysis of variance (ANOVA) followed by Tukey’s post hoc test. * *P* ≤ 0.05, * *P* ≤ 0.01, *** *P* ≤ 0.001.

**Additional file 4. Fig. S2**: Bioinformatic analysis of the lung transcriptome in CB, O_3_, and co-exposed mice. (**A**) Principal component analysis (PCA) illustrating the distribution of individual samples in each group. (**B**) Differential gene expression matrix, with the number and color correlating to the genes significantly altered between comparisons. (**C**) Heatmap of the top 500 differentially regulated genes between all groups. The heatmap displays the top differentially expressed genes by Padj-value, stratified by the Co-Exposure group on Day 4. We chose 500 as a conservative number to help capture more differentially expressed genes in other groups (ranging from ~ 200 to ~ 4000 differentially expressed genes, depending on group), while allowing for the graphic to still be interpretable. Day 1 (n = 4, each group) and Day 4 (n = 3, each group). Sham mice were exposed to filtered air. Number following the exposure condition denotes number of times (either one or four) animals were exposed. Animals were euthanized and analyzed 24 h post single or four exposures. Sham – 1 = filtered air exposed for 1 day, Sham – 4 = filtered air exposed for 4 days, CB – 1 = carbon black exposed (10 mg/m^3^) for a duration of (3 h) for 1 day, CB – 4 = carbon black exposed (10 mg/m^3^) for a duration of (3 h) for 4 days, O_3_ – 1 = ground level ozone exposed (2 ppm) for a duration of (3 h) for 1 day, O_3_ – 4 = ground level ozone exposed (2 ppm) for a duration of (3 h) for 4 days, CB-O_3_ – 1 = carbon black (10 mg/m^3^) and ground level ozone exposed (2 ppm) for a duration of (3 h) for 1 day, CB-O_3_ – 4 = carbon black (10 mg/m^3^) and ground level ozone exposed (2 ppm) for a duration of (3 h) for 4 days.

**Additional file 5. Fig. S3**: Volcano plots depicting changes in gene expression by comparison to the Sham exposure group. Genes depicted as green are not considered statistically different. Day 1 (n = 4, each group) and Day 4 (n = 3, each group). Sham – 1 = filtered air exposed for 1 day, Sham – 4 = filtered air exposed for 4 days, CB – 1 = carbon black exposed (10 mg/m^3^) for a duration of (3 h) for 1 day, CB – 4 = carbon black exposed (10 mg/m^3^) for a duration of (3 h) for 4 days, O_3_ – 1 = ground level ozone exposed (2 ppm) for a duration of (3 h) for 1 day, O_3_ – 4 = ground level ozone exposed (2 ppm) for a duration of (3 h) for 4 days, CB-O_3_ – 1 = carbon black (10 mg/m^3^) and ground level ozone exposed (2 ppm) for a duration of (3 h) for 1 day, CB-O_3_ – 4 = carbon black (10 mg/m^3^) and ground level ozone exposed (2 ppm) for a duration of (3 h) for 4 days, P = genes with -log_10_ *P*-adjusted value of > 1.3 and log_2_ fold change < 1, P & Log_2_FC = genes with -log_10_ *P*-adjusted value of > 1.3 and log_2_ fold change > 1.

**Additional file 6. Fig. S4**: Differential gene expression unique to the co-exposure groups. Venn diagrams for (**A**) single and (**B**) four exposures. The unique differentially expressed genes were placed in a volcano plot to highlight the expression profile for (**C**) single and (**D**) four exposures. Day 1 (n = 4, each group) and Day 4 (n = 3, each group). Sham mice were exposed to filtered air. Number following the exposure condition denotes number of times (either one or four) animals were exposed. Animals were euthanized and analyzed 24 h post single or four exposures. P = genes with -log_10_ *P*-adjusted value of > 1.3 and log_2_ fold change < 1, P & Log_2_FC = genes with -log_10_ *P*-adjusted value of > 1.3 and log_2_ fold change > 1, DE = differentially expressed genes.

**Additional file 7. Fig. S5**: Common ontology pathways between all groups. (**A**) 6-way Venn diagram illustrating the similar/dissimilar gene pathways between groups. (**B**) The three shared pathways between all groups are outlined with a few representative genes selected in the up regulated and down regulated columns. Sham – 1 = filtered air exposed for 1 day, Sham – 4 = filtered air exposed for 4 days, CB – 1 = carbon black exposed (10 mg/m^3^) for a duration of (3 h) for 1 day, CB – 4 = carbon black exposed (10 mg/m^3^) for a duration of (3 h) for 4 days, O_3_ – 1 = ground level ozone exposed (2 ppm) for a duration of (3 h) for 1 day, O_3_ – 4 = ground level ozone exposed (2 ppm) for a duration of (3 h) for 4 days, CB-O_3_ – 1 = carbon black (10 mg/m^3^) and ground level ozone exposed (2 ppm) for a duration of (3 h) for 1 day, CB-O_3_ – 4 = carbon black (10 mg/m^3^) and ground level ozone exposed (2 ppm) for a duration of (3 h) for 4 days, ID = ID of the enriched term for KEGG ontology, Term Description = description of the enriched term and identified pathway, Fold Enrichment = fold enrichment value for the enriched term (Calculated using ONLY the input genes), Up Regulated = genes that are increased in expression in the pathway, Down Regulated = genes that are decreased in expression in the pathway.

**Additional file 8. Fig. S6**: Examining mitochondrial protein content (**A**) Western blot analysis for a protein subunit of mitochondrial ETC complex I (NDUFA9), complex II (SDHA), complex III (UQCRC2), complex IV (COXIV) and complex V/ATP Synthase (ATP5A), normalized to VDAC expression. Data are presented as mean ± SEM of n = 3–4 mice per group and analyzed by two-way analysis of variance (ANOVA) followed by Tukey’s post hoc test. * *P* ≤ 0.05, * *P* ≤ 0.01, *** *P* ≤ 0.001. Sham – 4 = filtered air exposed for 4 days, CB – 4 = 10 mg/m^3^ CB exposure for 3 h repeated four times (24 h apart), O_3_ – 4 = 2 ppm O_3_ exposure for 3 h repeated four times (24 h apart), CB + O_3_ – 4 = 10 mg/m^3^ CB and 2 ppm O_3_ inhalation co-exposure for 3 h repeated four times (24 h apart), CI = complex I, CII = complex II, CIII = complex III, CIV = complex IV, CV = complex V.

**Additional file 9. Fig. S7**: Light photomicrographs of centriacinar regions in the lungs of mice exposed for 4 days to A) filtered air (controls), B) carbon black, C) ozone, and D) carbon black and ozone. Airway epithelium (e) lining terminal bronchioles (tb), alveolar type II epithelial cells and associated alveolar macrophages in proximal alveolar ducts (ad) are immunohistochemically stained for ATP5F1 in C) and D). Stippled arrows, carbon black particles. Tissues were counterstained with hematoxylin.

## Data Availability

All sequencing data and code have been made freely available: (1) Coding for bioinformatics is provided here: https://github.com/qahathaway/Co-Exposure; (2) Raw and processed transcriptomic reads have been added here: https://www.ncbi.nlm.nih.gov/geo/query/acc.cgi?acc=GSE161538; (3) Raw data values used for generation of heat maps for Real time PCR/ ELISA, and primer sequences are provided in Additional file 1.(4) Differential expression analysis is provided in Additional file 2; (5) Other data sets analyzed in this study are available from corresponding author on reasonable request.
