# Supplementary figures and images for: Transcriptomics of single dose and repeated carbon black and ozone inhalation co-exposure highlight progressive pulmonary mitochondrial dysfunction
Source: Part Fibre Toxicol. 2021 Dec 15;18:44. doi: 10.1186/s12989-021-00437-8 (PMC8672524; doi:10.1186/s12989-021-00437-8)

## Slide 1
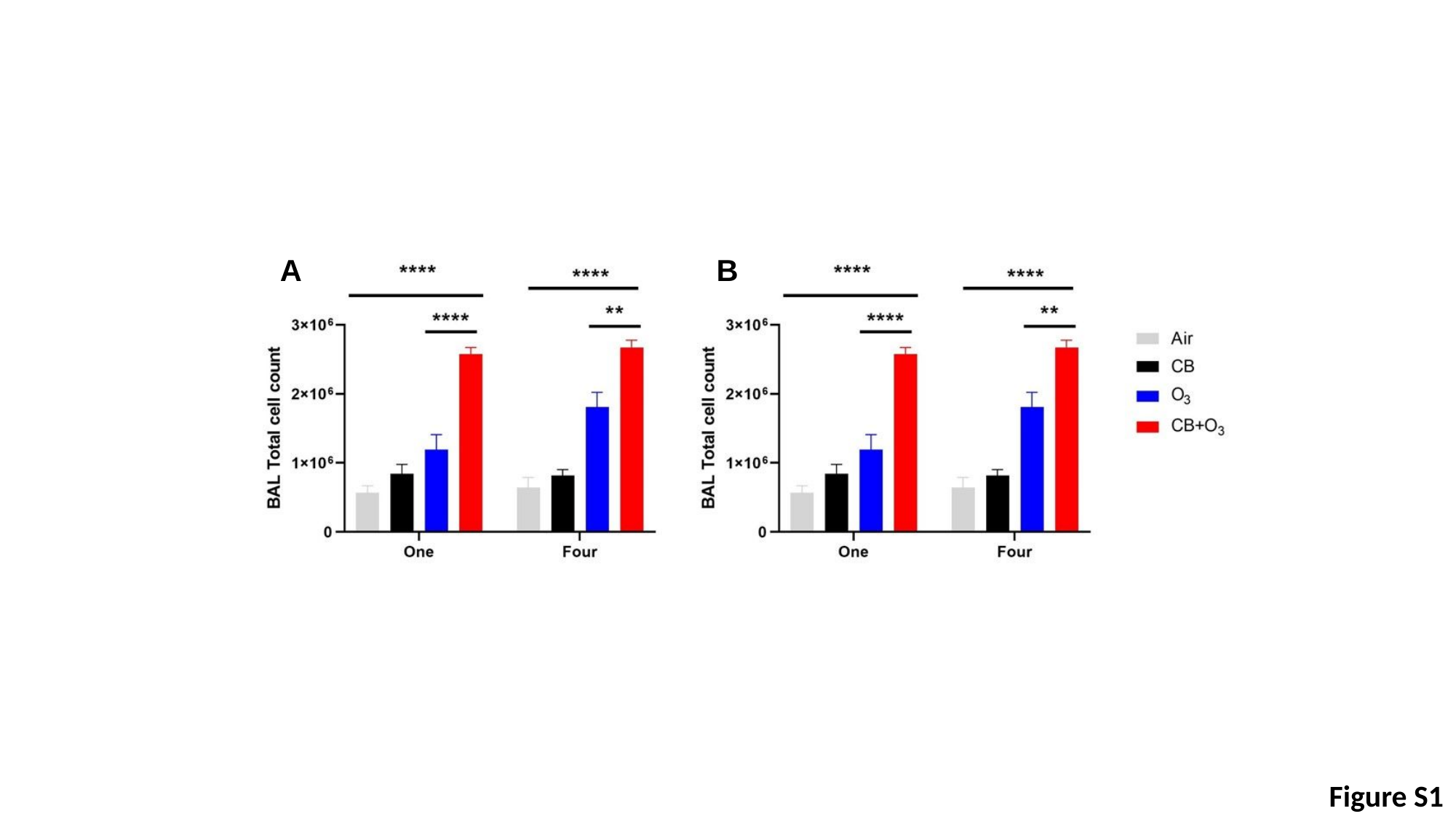

A
B
Figure S1

Supplement: Supplementary file 3 — Additional file 3. Fig. S1: Bronchoalveolar lavage total cells and macrophages depicting increased potency of CB + O3 co-exposure to induce lung inflammation after (A) 1 Day exposure (n = 5–7) and (B) 4 Days exposure (n = 5–7) compared to filtered air, CB (10 mg/m3) and O3 (2 ppm). Data are presented as mean ± SEM of n = 5–7 mice per group and analyzed by two-way analysis of variance (ANOVA) followed by Tukey’s post hoc test. * P ≤ 0.05, * P ≤ 0.01, *** P ≤ 0.001. [file 12989_2021_437_MOESM3_ESM.pptx]

## Slide 1
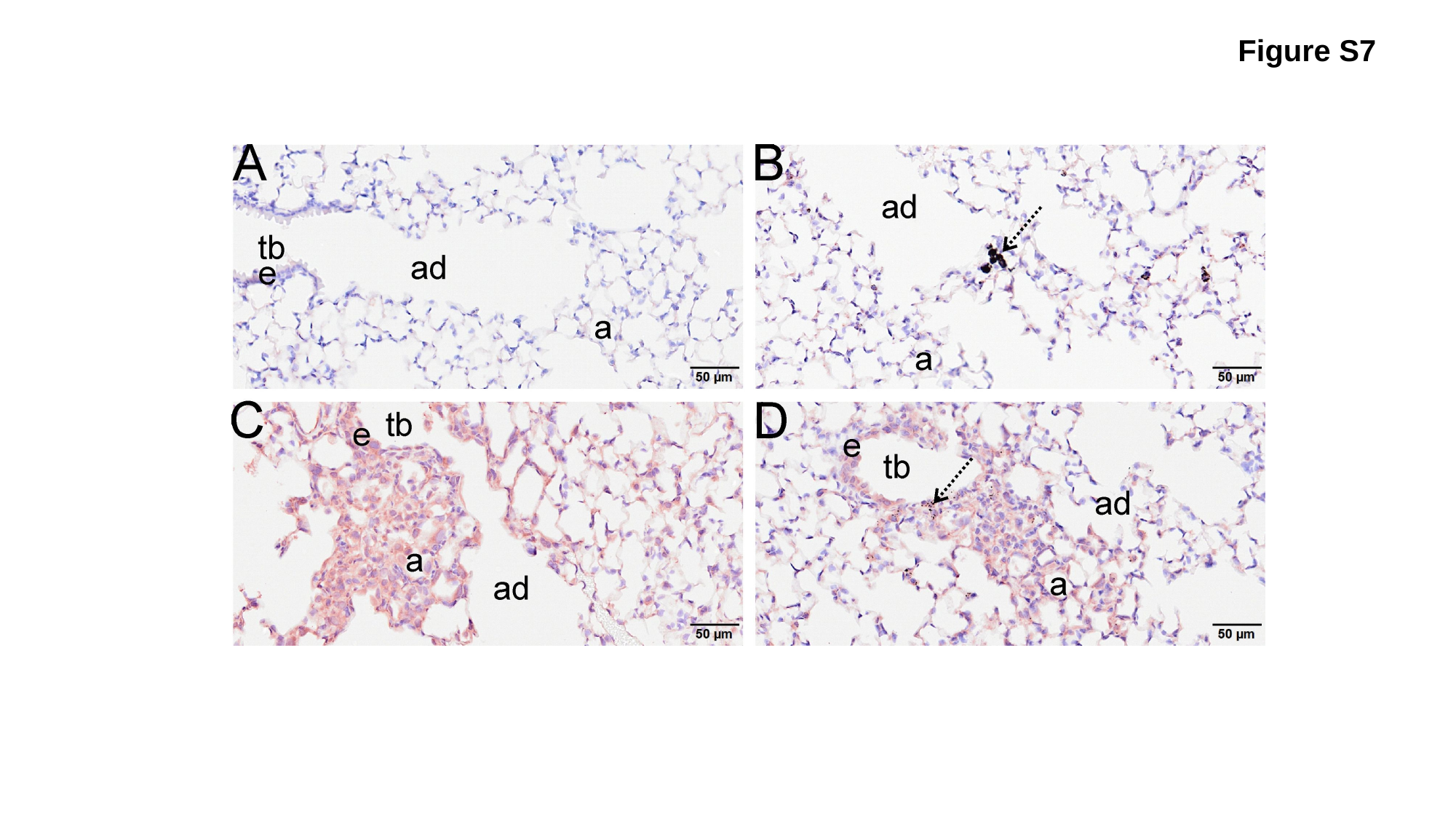

Figure S7

Supplement: Supplementary file 9 — Additional file 9. Fig. S7: Light photomicrographs of centriacinar regions in the lungs of mice exposed for 4 days to A) filtered air (controls), B) carbon black, C) ozone, and D) carbon black and ozone. Airway epithelium (e) lining terminal bronchioles (tb), alveolar type II epithelial cells and associated alveolar macrophages in proximal alveolar ducts (ad) are immunohistochemically stained for ATP5F1 in C) and D). Stippled arrows, carbon black particles. Tissues were counterstained with hematoxylin. [file 12989_2021_437_MOESM9_ESM.pptx]
