# Supplementary material for: Transcriptomics of single dose and repeated carbon black and ozone inhalation co-exposure highlight progressive pulmonary mitochondrial dysfunction
Source: Part Fibre Toxicol. 2021 Dec 15;18:44. doi: 10.1186/s12989-021-00437-8 (PMC8672524; doi:10.1186/s12989-021-00437-8)
Supplement: Supplementary file 4 — Additional file 4. Fig. S2: Bioinformatic analysis of the lung transcriptome in CB, O3, and co-exposed mice. (A) Principal component analysis (PCA) illustrating the distribution of individual samples in each group. (B) Differential gene expression matrix, with the number and color correlating to the genes significantly altered between comparisons. (C) Heatmap of the top 500 differentially regulated genes between all groups. The heatmap displays the top differentially expressed genes by Padj-value, stratified by the Co-Exposure group on Day 4. We chose 500 as a conservative number to help capture more differentially expressed genes in other groups (ranging from ~ 200 to ~ 4000 differentially expressed genes, depending on group), while allowing for the graphic to still be interpretable. Day 1 (n = 4, each group) and Day 4 (n = 3, each group). Sham mice were exposed to filtered air. Number following the exposure condition denotes number of times (either one or four) animals were exposed. Animals were euthanized and analyzed 24 h post single or four exposures. Sham – 1 = filtered air exposed for 1 day, Sham – 4 = filtered air exposed for 4 days, CB – 1 = carbon black exposed (10 mg/m3) for a duration of (3 h) for 1 day, CB – 4 = carbon black exposed (10 mg/m3) for a duration of (3 h) for 4 days, O3 – 1 = ground level ozone exposed (2 ppm) for a duration of (3 h) for 1 day, O3 – 4 = ground level ozone exposed (2 ppm) for a duration of (3 h) for 4 days, CB-O3 – 1 = carbon black (10 mg/m3) and ground level ozone exposed (2 ppm) for a duration of (3 h) for 1 day, CB-O3 – 4 = carbon black (10 mg/m3) and ground level ozone exposed (2 ppm) for a duration of (3 h) for 4 days. [file 12989_2021_437_MOESM4_ESM.pptx]

## Slide 1
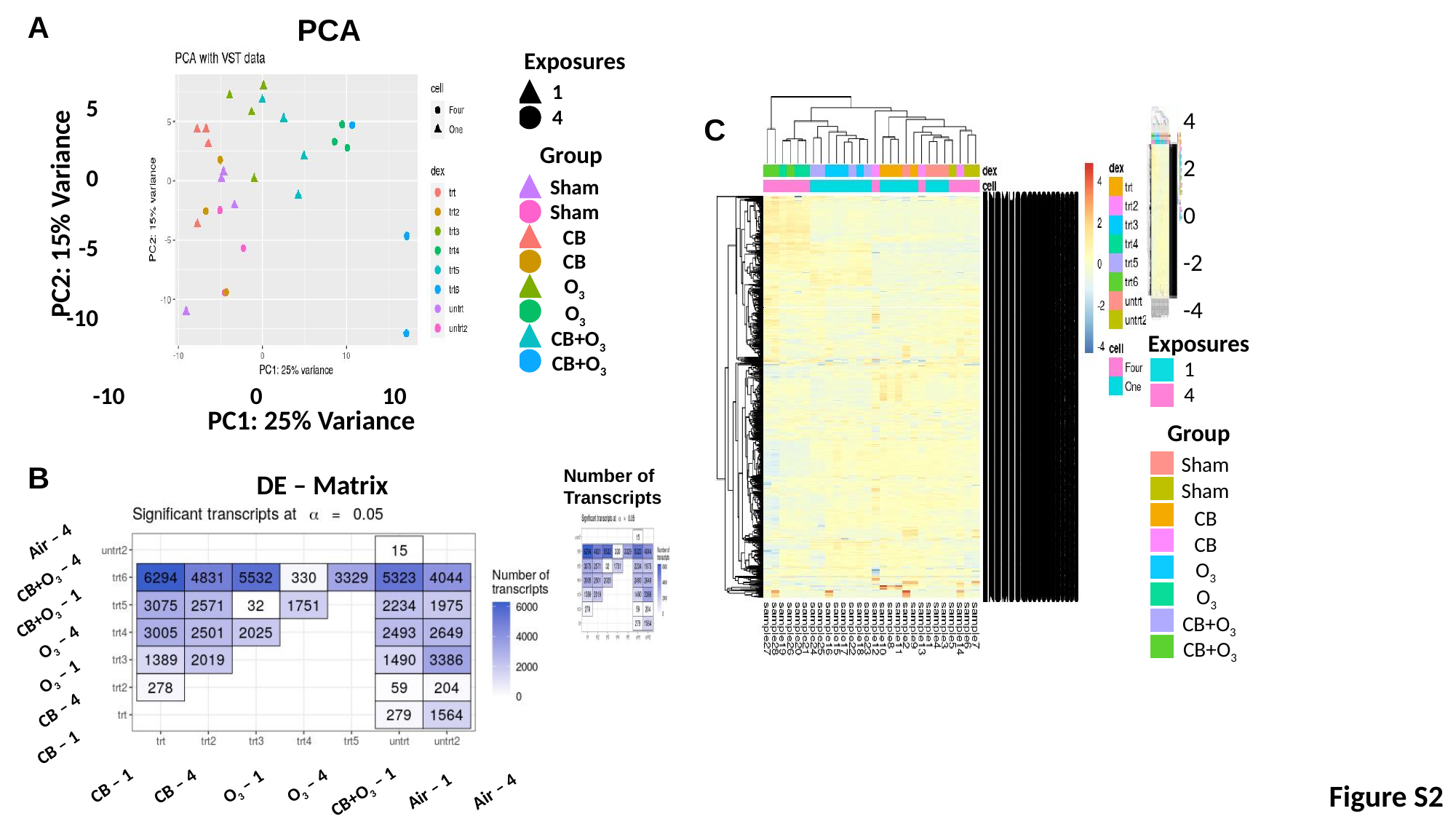

A
PCA
Exposures
1
4
Group
Sham
Sham
CB
CB
O3
O3
CB+O3
CB+O3
5
0
-5
-10
PC2: 15% Variance
-10 0 10
PC1: 25% Variance
4
2
0
-2
-4
C
Exposures
1
4
Group
Sham
B
Number of
Transcripts
DE – Matrix
Sham
CB
Air – 4
CB
O3
CB+O3 – 4
O3
CB+O3 – 1
CB+O3
O3 – 4
CB+O3
O3 – 1
CB – 4
CB – 1
CB – 1
O3 – 4
CB – 4
O3 – 1
Figure S2
Air – 1
Air – 4
CB+O3 – 1
