# Supplementary material for: Transcriptomics of single dose and repeated carbon black and ozone inhalation co-exposure highlight progressive pulmonary mitochondrial dysfunction
Source: Part Fibre Toxicol. 2021 Dec 15;18:44. doi: 10.1186/s12989-021-00437-8 (PMC8672524; doi:10.1186/s12989-021-00437-8)
Supplement: Supplementary file 5 — Additional file 5. Fig. S3: Volcano plots depicting changes in gene expression by comparison to the Sham exposure group. Genes depicted as green are not considered statistically different. Day 1 (n = 4, each group) and Day 4 (n = 3, each group). Sham – 1 = filtered air exposed for 1 day, Sham – 4 = filtered air exposed for 4 days, CB – 1 = carbon black exposed (10 mg/m3) for a duration of (3 h) for 1 day, CB – 4 = carbon black exposed (10 mg/m3) for a duration of (3 h) for 4 days, O3 – 1 = ground level ozone exposed (2 ppm) for a duration of (3 h) for 1 day, O3 – 4 = ground level ozone exposed (2 ppm) for a duration of (3 h) for 4 days, CB-O3 – 1 = carbon black (10 mg/m3) and ground level ozone exposed (2 ppm) for a duration of (3 h) for 1 day, CB-O3 – 4 = carbon black (10 mg/m3) and ground level ozone exposed (2 ppm) for a duration of (3 h) for 4 days, P = genes with -log10 P-adjusted value of > 1.3 and log2 fold change < 1, P & Log2FC = genes with -log10 P-adjusted value of > 1.3 and log2 fold change > 1. [file 12989_2021_437_MOESM5_ESM.pptx]

## Slide 1
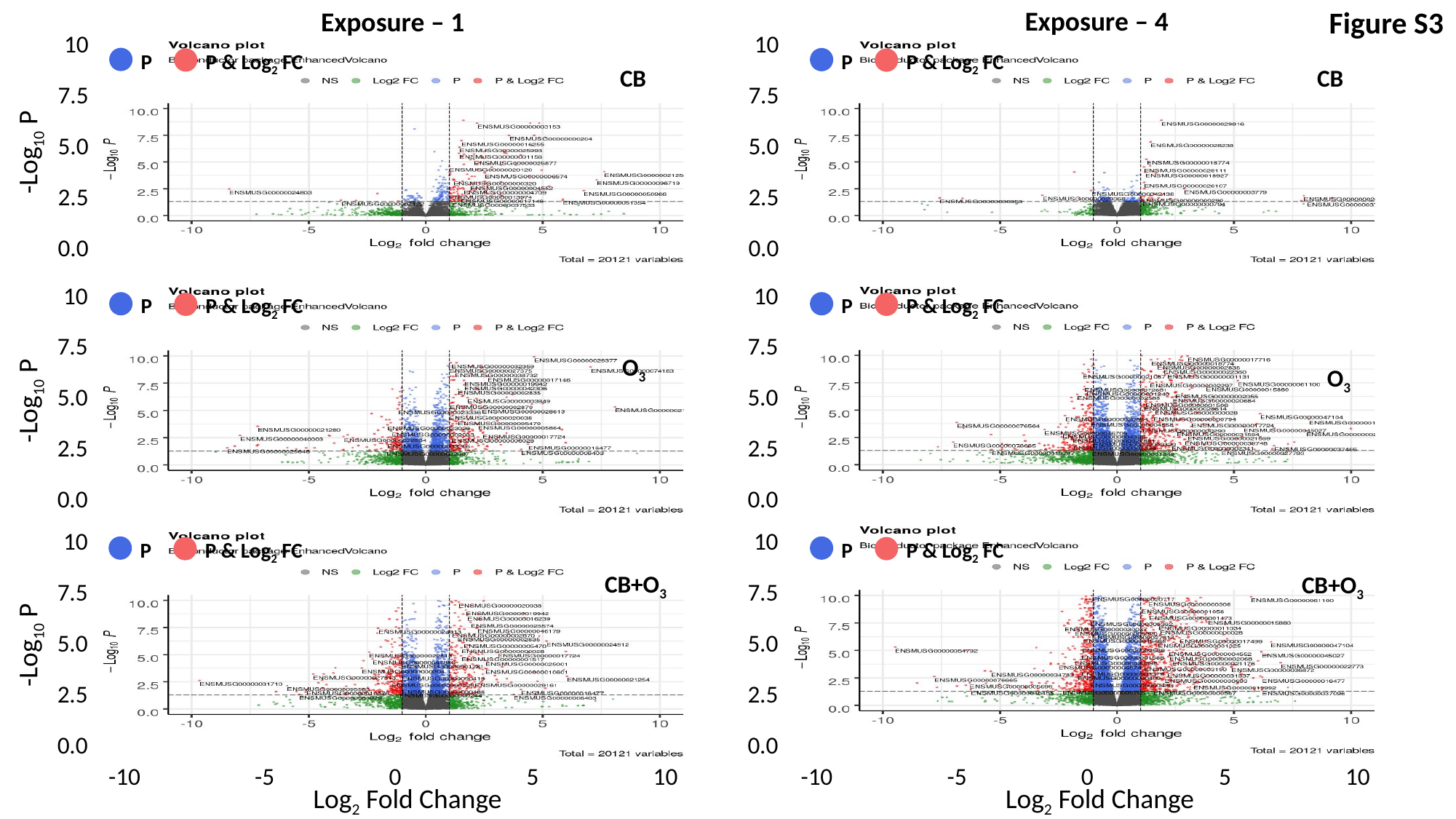

Figure S3
Exposure – 4
Exposure – 1
10
7.5
5.0
2.5
0.0
10
7.5
5.0
2.5
0.0
P
P & Log2 FC
P
P & Log2 FC
CB
CB
-Log10 P
10
7.5
5.0
2.5
0.0
10
7.5
5.0
2.5
0.0
P
P & Log2 FC
P
P & Log2 FC
O3
O3
-Log10 P
10
7.5
5.0
2.5
0.0
10
7.5
5.0
2.5
0.0
P
P & Log2 FC
P
P & Log2 FC
CB+O3
CB+O3
-Log10 P
-10 -5 0 5 10
-10 -5 0 5 10
Log2 Fold Change
Log2 Fold Change
