# Supplementary material for: Transcriptomics of single dose and repeated carbon black and ozone inhalation co-exposure highlight progressive pulmonary mitochondrial dysfunction
Source: Part Fibre Toxicol. 2021 Dec 15;18:44. doi: 10.1186/s12989-021-00437-8 (PMC8672524; doi:10.1186/s12989-021-00437-8)
Supplement: Supplementary file 6 — Additional file 6. Fig. S4: Differential gene expression unique to the co-exposure groups. Venn diagrams for (A) single and (B) four exposures. The unique differentially expressed genes were placed in a volcano plot to highlight the expression profile for (C) single and (D) four exposures. Day 1 (n = 4, each group) and Day 4 (n = 3, each group). Sham mice were exposed to filtered air. Number following the exposure condition denotes number of times (either one or four) animals were exposed. Animals were euthanized and analyzed 24 h post single or four exposures. P = genes with -log10 P-adjusted value of > 1.3 and log2 fold change < 1, P & Log2FC = genes with -log10 P-adjusted value of > 1.3 and log2 fold change > 1, DE = differentially expressed genes. [file 12989_2021_437_MOESM6_ESM.pptx]

## Slide 1
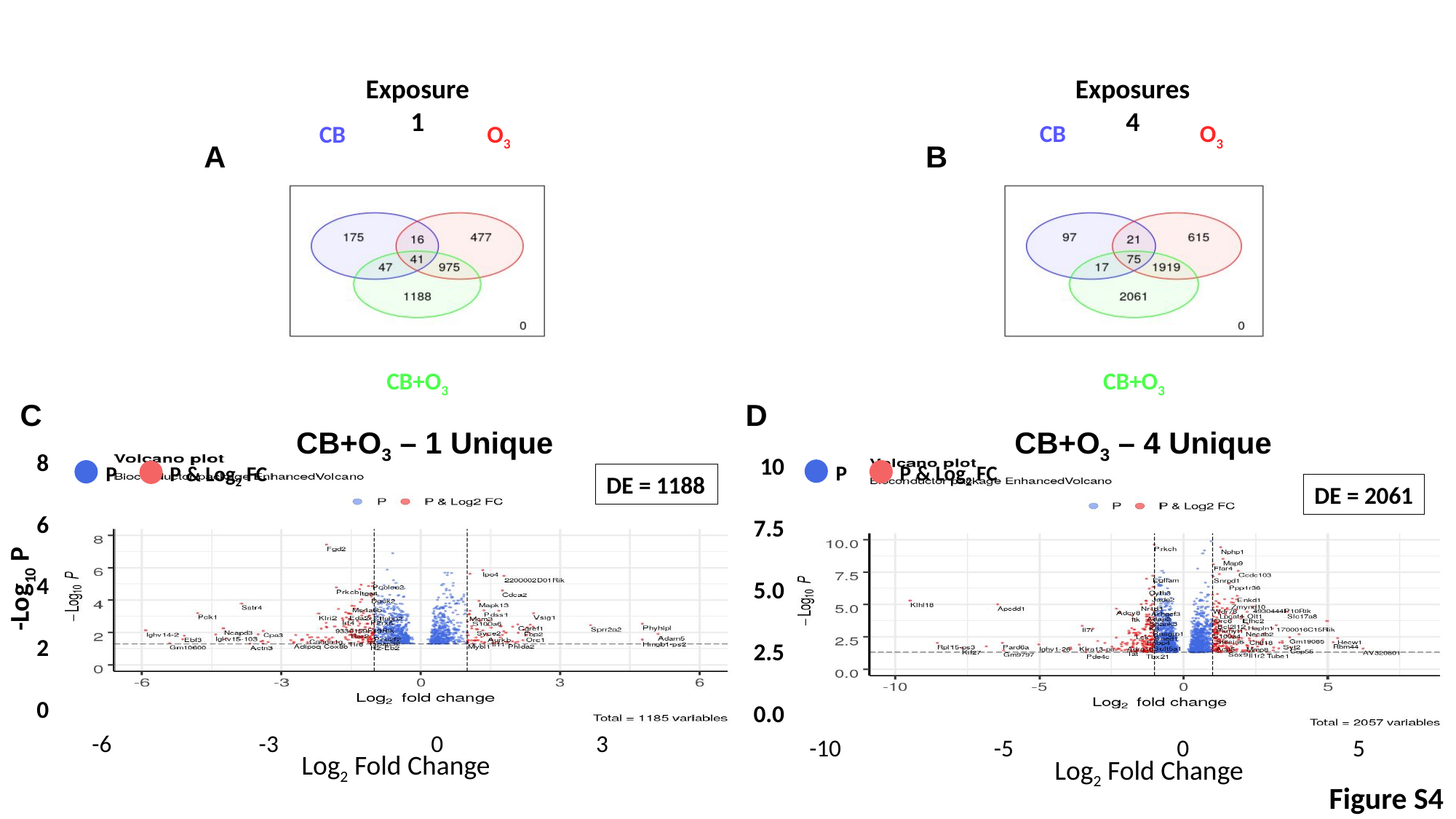

Exposure
1
Exposures
4
CB
O3
CB
O3
A
B
CB+O3
CB+O3
C
D
CB+O3 – 1 Unique
CB+O3 – 4 Unique
8
6
4
2
0
-Log10 P
-6 -3 0 3
10
7.5
5.0
2.5
0.0
DE = 2061
-10 -5 0 5
P
P & Log2 FC
P
P & Log2 FC
DE = 1188
Log2 Fold Change
Log2 Fold Change
Figure S4
