# Supplementary material for: Transcriptomics of single dose and repeated carbon black and ozone inhalation co-exposure highlight progressive pulmonary mitochondrial dysfunction
Source: Part Fibre Toxicol. 2021 Dec 15;18:44. doi: 10.1186/s12989-021-00437-8 (PMC8672524; doi:10.1186/s12989-021-00437-8)
Supplement: Supplementary file 7 — Additional file 7. Fig. S5: Common ontology pathways between all groups. (A) 6-way Venn diagram illustrating the similar/dissimilar gene pathways between groups. (B) The three shared pathways between all groups are outlined with a few representative genes selected in the up regulated and down regulated columns. Sham – 1 = filtered air exposed for 1 day, Sham – 4 = filtered air exposed for 4 days, CB – 1 = carbon black exposed (10 mg/m3) for a duration of (3 h) for 1 day, CB – 4 = carbon black exposed (10 mg/m3) for a duration of (3 h) for 4 days, O3 – 1 = ground level ozone exposed (2 ppm) for a duration of (3 h) for 1 day, O3 – 4 = ground level ozone exposed (2 ppm) for a duration of (3 h) for 4 days, CB-O3 – 1 = carbon black (10 mg/m3) and ground level ozone exposed (2 ppm) for a duration of (3 h) for 1 day, CB-O3 – 4 = carbon black (10 mg/m3) and ground level ozone exposed (2 ppm) for a duration of (3 h) for 4 days, ID = ID of the enriched term for KEGG ontology, Term Description = description of the enriched term and identified pathway, Fold Enrichment = fold enrichment value for the enriched term (Calculated using ONLY the input genes), Up Regulated = genes that are increased in expression in the pathway, Down Regulated = genes that are decreased in expression in the pathway. [file 12989_2021_437_MOESM7_ESM.pptx]

## Slide 1
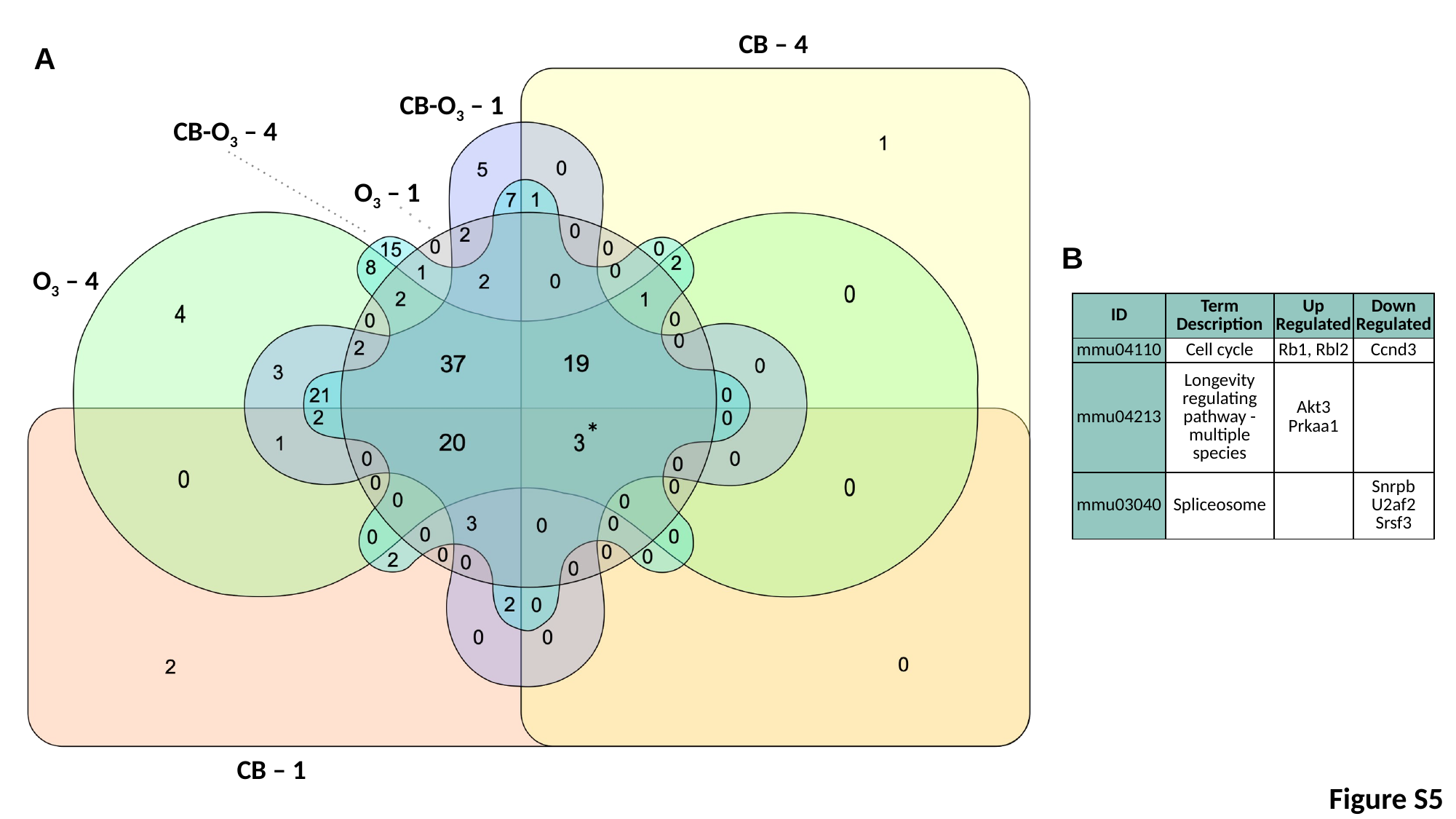

CB – 4
A
CB-O3 – 1
CB-O3 – 4
O3 – 1
B
O3 – 4
| ID | Term Description | Up Regulated | Down Regulated |
| --- | --- | --- | --- |
| mmu04110 | Cell cycle | Rb1, Rbl2 | Ccnd3 |
| mmu04213 | Longevity regulating pathway - multiple species | Akt3 Prkaa1 | |
| mmu03040 | Spliceosome | | Snrpb U2af2 Srsf3 |
*
CB – 1
Figure S5
