# Supplementary material for: Transcriptomics of single dose and repeated carbon black and ozone inhalation co-exposure highlight progressive pulmonary mitochondrial dysfunction
Source: Part Fibre Toxicol. 2021 Dec 15;18:44. doi: 10.1186/s12989-021-00437-8 (PMC8672524; doi:10.1186/s12989-021-00437-8)
Supplement: Supplementary file 8 — Additional file 8. Fig. S6: Examining mitochondrial protein content (A) Western blot analysis for a protein subunit of mitochondrial ETC complex I (NDUFA9), complex II (SDHA), complex III (UQCRC2), complex IV (COXIV) and complex V/ATP Synthase (ATP5A), normalized to VDAC expression. Data are presented as mean ± SEM of n = 3–4 mice per group and analyzed by two-way analysis of variance (ANOVA) followed by Tukey’s post hoc test. * P ≤ 0.05, * P ≤ 0.01, *** P ≤ 0.001. Sham – 4 = filtered air exposed for 4 days, CB – 4 = 10 mg/m3 CB exposure for 3 h repeated four times (24 h apart), O3 – 4 = 2 ppm O3 exposure for 3 h repeated four times (24 h apart), CB + O3 – 4 = 10 mg/m3 CB and 2 ppm O3 inhalation co-exposure for 3 h repeated four times (24 h apart), CI = complex I, CII = complex II, CIII = complex III, CIV = complex IV, CV = complex V. [file 12989_2021_437_MOESM8_ESM.pptx]

## Slide 1
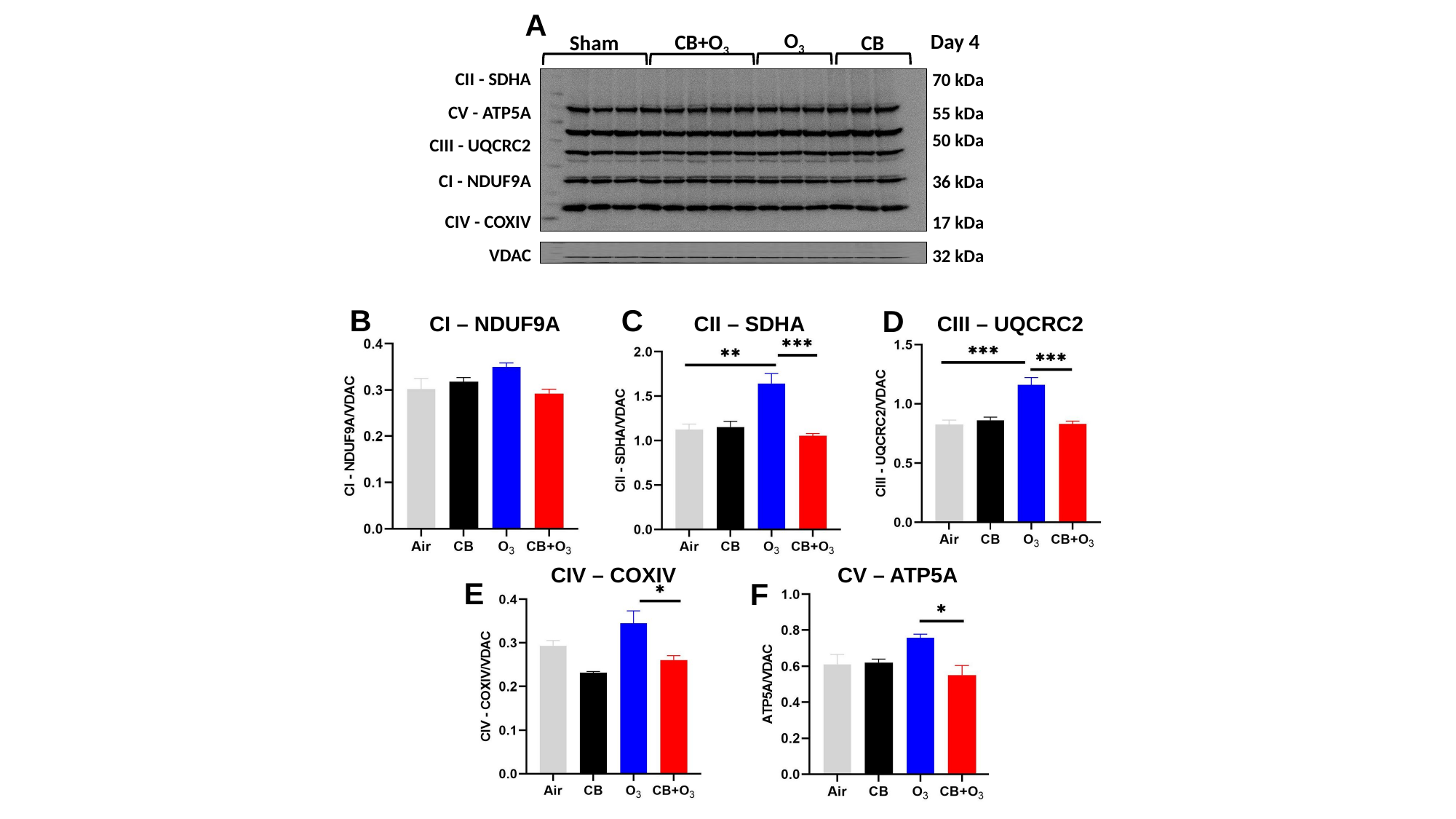

A
O3
Day 4
CB+O3
CB
Sham
CII - SDHA
70 kDa
CV - ATP5A
55 kDa
50 kDa
CIII - UQCRC2
CI - NDUF9A
36 kDa
CIV - COXIV
17 kDa
VDAC
32 kDa
C
B
D
CII – SDHA
CIII – UQCRC2
CI – NDUF9A
CV – ATP5A
CIV – COXIV
E
F
